# Downregulation of GJA1-Derived circ_753 and circ_755 Differentially Modulates miR-182-5p Expression and Breast Cancer-Associated Phenotypes

**DOI:** 10.3390/ijms27198849

**Published:** 2026-10-03

**Authors:** Nour Maatouk, Joséphine Briand, Jana Ismael, Serena Andraos, Abdallah Kurdi, Sophie Lelièvre, Rihab Nasr, Rabih Talhouk

**Affiliations:** 1 Department of Biology, Faculty of Arts and Sciences, American University of Beirut, Beirut 1107 2020, Lebanon; nam43@mail.aub.edu (N.M.); jsi03@mail.aub.edu (J.I.); serena.andraos3@gmail.com (S.A.); 2 Institut de Cancérologie de l’Ouest, 49055 Angers, France; josephine.briand@ico.unicancer.fr (J.B.); sophie.lelievre@ico.unicancer.fr (S.L.); 3 Department of Biochemistry and Molecular Genetics, Faculty of Medicine, American University of Beirut, Beirut 1107 2020, Lebanon; ak161@aub.edu.lb; 4 Department of Anatomy, Cell Biology and Physiological Sciences, Faculty of Medicine, American University of Beirut, Beirut 1107 2020, Lebanon; rn03@aub.edu.lb

**Keywords:** *GJA1* (Connexin 43), circular RNA (circRNA), microRNA (miRNA), breast epithelial cells, early breast cancer initiation, three-dimensional (3D) culture, extracellular vesicles (EVs)

## Abstract

CircRNAs are key regulators of gene expression. The functional roles of exonic versus exon–intron circRNAs in early breast cancer remain unclear. Connexin-43 (*GJA1*) generates exonic circRNA (ecircRNA) hsa_circ_0077755 (circ_755) and exon–intron circRNA (EIcircRNA), hsa_circ_0077753 (circ_753), both linked to increased breast cancer risk. We examined their structural, molecular, and phenotypic roles in transient and stable knockdown S1 breast epithelial cells, using RT-qPCR, dual-luciferase, lumen formation and invasion assays, and extracellular vesicle (EV) analysis. Knockdown of cytoplasm-enriched circ_755 reduced Connexin-43 transcript and protein, whereas knockdown of nuclear-enriched circ_753 preserved *GJA1*. Both induced lumen disruption, β-catenin disorganization, increased invasion, and regulated onco-miR-182. More pronounced miR-182 upregulation and broader repression of tumor-suppressive targets (*BCL2, FOXO1, RECK*) were observed in circ_753 knockdown cells, alongside concurrent depletion of hsa_circ_0008932 (circ_8932), which is predicted to interact with miR-182. Similar reciprocal changes in circRNA/miR-182 were observed in released EVs. These data highlight circRNA-miR-182 networks with distinct effects on tumor initiation and potential value as breast cancer biomarkers.

## 1. Introduction

Breast cancer is the second most prevalent cancer after lung cancer worldwide and is the most frequently diagnosed, yet it remains the leading cause of cancer-related deaths among women [1]. Growing evidence highlights the importance of co- and post-transcriptional RNA molecules, such as circular RNA (circRNAs) and microRNAs (miRNAs), in regulating gene expression. CircRNAs represent a distinct class of evolutionarily conserved noncoding RNAs generated through back-splicing events, resulting in covalently closed-loop structures that lack both 5′ caps and 3′ poly(A) tails [2]. This unique characteristic renders them highly stable compared to linear RNA transcripts, allowing them to participate in diverse cellular regulatory processes. Advances in high-throughput sequencing and bioinformatic analyses have led to the identification of thousands of circRNAs that exhibit tissue- and cancer-specific expression [3,4,5]. CircRNAs are aberrantly expressed in a wide range of human cancers, including BC, where they contribute to tumor initiation and progression. A single gene locus can produce multiple circular RNA isoforms via alternative back-splicing and internal alternative splicing. Each isoform has a unique exon composition and intron retention. Such structural variations might influence their subcellular localization, molecular interactions, and regulatory function. Fully exonic circRNAs (ecircRNAs) are frequently detected in the cytoplasmic compartment. They can participate in post-transcriptional regulatory processes, whereas exon–intron circRNAs (EIciRNAs) are often enriched in the nucleus, where some have been shown to interact with U1 snRNP and RNA polymerase II and regulate transcription. Thus, circRNAs originating from the same parental gene should not necessarily be assumed to be functionally redundant, providing a rationale for isoform-level investigation. Research indicates that circRNAs can inhibit the function of miRNA by acting as a miRNA sponge [6,7]. MiRNAs are a class of small noncoding RNAs that play a robust role in regulating gene expression at the post-transcriptional level, essentially by promoting mRNA degradation or inhibiting mRNA translation [8,9]. MiRNAs can regulate up to 200 downstream target transcripts, thereby influencing entire signaling pathways and complex cellular behaviors [10,11]. In addition to their canonical role in mRNA regulation, miRNAs have also been shown to interact with other classes of noncoding RNAs, including long noncoding RNAs and circRNAs, thereby expanding their functional effects within the transcriptome [12,13]. Dysregulation of miRNA expression is a hallmark of many human diseases, including cancer, where the altered miRNome contributes to genome instability, aberrant signaling, and malignant resistance and progression [14].

We have previously proposed connexin 43 (Cx43)/hsa_circ_0077755/miR-182-5p (miR-182) as a potential biomarker signature axis for heightened risk of breast cancer initiation [15]. Accordingly, loss of Cx43 expression in the breast epithelium is associated with cell multilayering and polarity disruption, as well as loss of hsa_circ_0077755 (circ_755) expression, one of the Cx43-derived circRNAs. Consequently, the expression of hsa_miR-182, proposed to be sponged by circ_755, increases, ultimately affecting the expression of miR-182-regulated downstream pathways. Although Cx43 is proposed as an essential regulator in human breast epithelial tumorigenesis, the functional differences between Cx43-derived circRNAs, the EIcircRNA hsa_circ_0077753 (circ_753), and the fully exonic circ_755 isoforms derived from the same parental gene, *GJA1*, remain poorly understood.

This study investigated whether the two structurally distinct *GJA1*-derived circRNAs, circ_753 and circ_755, differentially contribute to the regulation of breast epithelial homeostasis and tumor-initiating phenotypes. Using the non-neoplastic human mammary epithelial S1 model, we examined the consequences of targeting each circRNA on *GJA1*/Cx43 expression, the miR-182-associated regulatory network, epithelial organization in three-dimensional culture, and invasive behavior. By comparing an exon–intron and a fully exonic circRNA originating from the same parental locus, we sought to determine whether *GJA1*-derived circRNAs exhibit nonredundant regulatory effects during early breast cancer-associated alterations.

## 2. Results

### 2.1. Selective siRNA-Mediated Silencing of Cx43-Derived circRNAs circ_753 and circ_755 and the Resulting Effect on GJA1 Expression in S1 Breast Epithelial Cells

**Figure 1:**
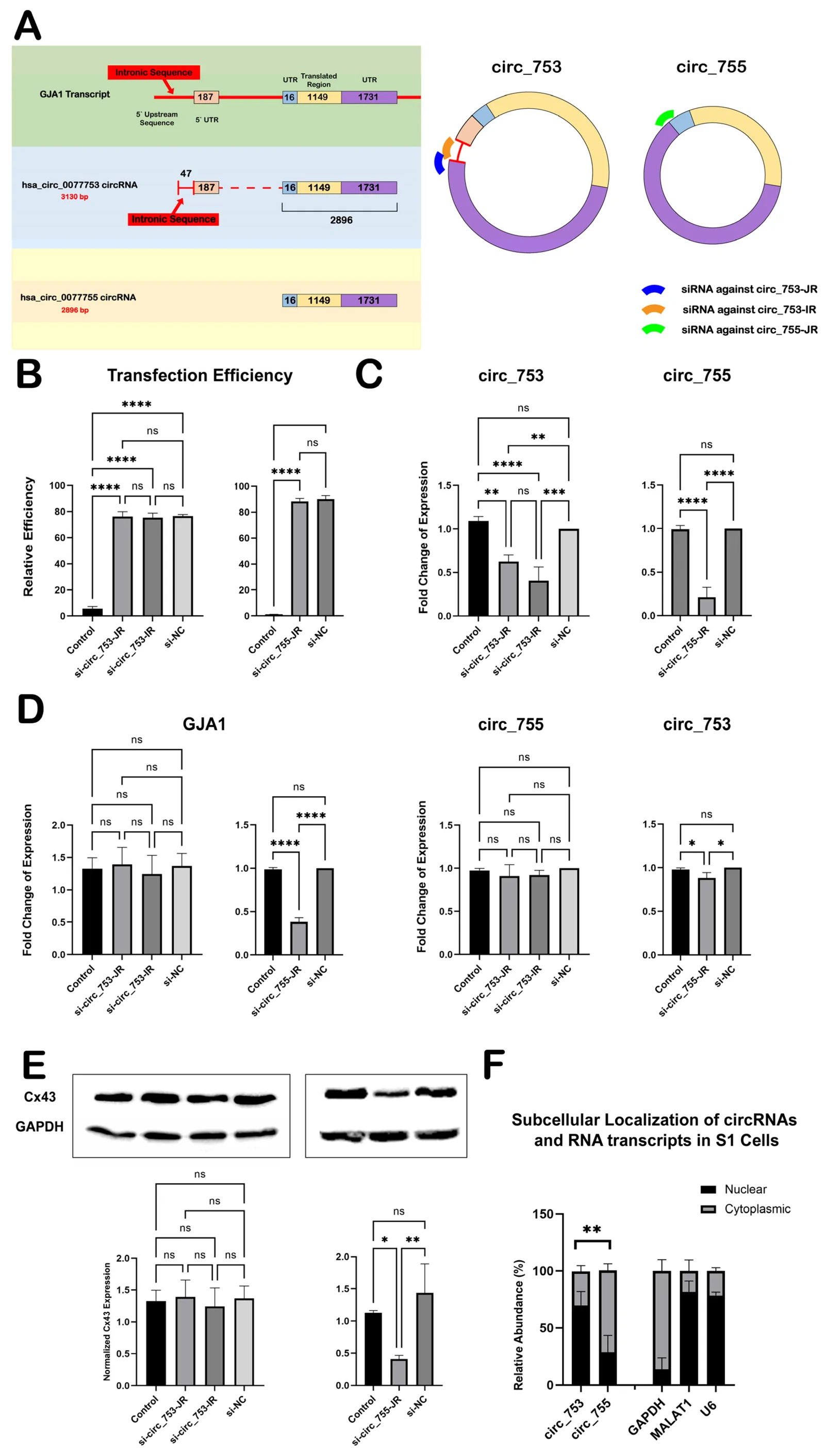
Differential effect on the host gene *GJA1*: EIciRNA hsa_circ_0077753 (circ_753) knockdown does not lower *GJA1*/Cx43 expression, whereas knockdown of EcircRNA hsa_circ_0077755 (circ_755) does. S1 cells were transfected with siRNAs as described, and all analyses were performed 24 h post-transfection. (**A**) Gene maps and siRNA design. Linear schematic of the human *GJA1* transcript (exons and intronic sequence) alongside diagrams of intron-exonic circ_753 and exonic circ_755. Colored ticks indicate siRNA target sites. JR = junctional region (siRNAs directed to the back-splice junction); IR = intronic region (siRNA directed to the intronic sequence unique to circ_753). Divergent qPCR primers for circRNAs span the back-splice junction; mRNA (*GJA1*) primers are conventional. (**B**) Bar plots of relative transfection efficiency (mean ± SEM, *n* = 3 biologically independent experiments), calculated as the percentage of GFP-positive cells in gate R2. Left: circ_753 experiment set (Control, si-circ_753-JR, si-circ_753-IR, si-NC). Right: circ_755 experiment set (Control, si-circ_755-JR, si-NC). (**C**) RT-qPCR assessment of circRNA knockdown efficiency. Bar graphs display relative expression on the y-axis as fold change (2^−ΔΔCt^), with 18S as the internal control and si-NC set to 1.0. Left: circ_753 expression following transfection with si-circ_753-JR or si-circ_753-IR. Right: circ_755 expression following transfection with si-circ_755-JR. Bars show mean ± SEM (*n* = 3 biologically independent experiments). (**D**) RT-qPCR assessment of *GJA1* expression and cross-isoform effects following circRNA knockdown. The left graphs show *GJA1* mRNA expression following circ_753 knockdown with si-circ_753-JR or si-circ_753-IR and following circ_755 knockdown with si-circ_755-JR, respectively. The right graphs show expression of the reciprocal circRNA to assess effects of each siRNA on the other *GJA1*-derived circular isoform: circ_755 expression following circ_753 knockdown and circ_753 expression following circ_755 knockdown. Relative expression is presented as fold change (2^−ΔΔCt^), with 18S as the internal control and si-NC set to 1.0. Bars represent mean ± SEM (*n* = 3 biologically independent experiments). (**E**) Cx43 protein expression by immunoblot. Top: representative Western blots for Cx43, with GAPDH as the loading control, following circ_753 knockdown (Control, si-circ_753-JR, si-circ_753-IR, si-NC) and circ_755 knockdown (Control, si-circ_755-JR, si-NC). Bottom: densitometric quantification of Cx43 expression normalized to GAPDH (mean ± SEM, *n* = 3 biologically independent experiments). (**F**) Subcellular localization of circRNAs and control transcripts in S1 cells. Nuclear and cytoplasmic fractions were isolated from S1 cells, and the relative abundance of circ_753, circ_755, GAPDH, *MALAT1*, and U6 was quantified by RT-qPCR. Bar graphs display the percentage distribution of each transcript between nuclear and cytoplasmic compartments, normalized such that the combined abundance across both fractions equals 100% for each biological replicate. circ_753 showed predominant nuclear localization, whereas circ_755 was enriched in the cytoplasmic fraction. GAPDH served as a cytoplasmic control, while *MALAT1* and U6 served as nuclear controls, confirming successful fractionation. Bars represent mean ± SEM (*n* = 3 biologically independent experiments). Controls and nomenclature used throughout: Control = untreated cells; si-NC = non-targeting negative-control siRNA; si-circ_753-JR/IR and si-circ_755-JR = siRNAs targeting the indicated junctional or intronic regions as defined in panel A. Significance notation: ns, not significant (*p* ≥ 0.05); * *p* < 0.05; ** *p* < 0.01; *** *p* < 0.001; **** *p* < 0.0001.

We first used CircInteractome to identify Cx43-derived circRNAs and then mapped their sequences to the corresponding Cx43 host nuclear RNA (hnRNA). circ_753 contained both exonic and intronic regions, whereas circ_755 was confirmed to be completely exonic (Figure 1A). Each circRNA has a distinct back-splice junction, which is why siRNAs were designed against the junctional region of each circRNA to ensure silencing. Furthermore, siRNA targeting the intronic region of circ_753 was designed to specifically target the intronic sequence of circ_753 (Figure 1A). The effectiveness of transient transfection (Figure 1B and Figure S1A) and specificity in silencing circRNAs of interest in S1 breast epithelial cells was demonstrated, with circ_753 expression reduced to ~0.6- and 0.4-fold of control levels with the siRNA targeting the junctional region (circ_753-JR) and intronic region (circ_753-IR), respectively (Figure 1C). Transient transfection of circ_755 revealed a 0.8-fold reduction in expression (Figure 1C). While circ_753 knockdown had no significant effect on parental gene expression, reduction in circ_755 expression was associated with a 0.6-fold reduction in Cx43 mRNA levels. Further cross-silencing assays showed that circ_755 silencing caused a slight reduction in circ_753 expression, but circ_753 silencing had no effect on circ_755 expression, suggesting that the regulatory effect of circ_753 and circ_755 on each other is not reciprocal (Figure 1D). Western blot analysis indicated that knockdown of circ_755 decreased Cx43 protein expression by 0.6-fold. Conversely, knockdown of circ_753 did not significantly affect Cx43 protein levels (Figure 1E). Taken together, these results demonstrated a functional correlation between the expression of Cx43, exonic circ_755, and EIcircRNA circ_753 at the transcript and protein levels, and importantly, that circ_753 does not influence its parent gene despite being derived from the same hnRNA. Since circ_753 contains intronic sequences, whereas circ_755 is fully exonic, we also examined whether these two circRNAs differ in their cellular localization. Nuclear and cytoplasmic fractions were prepared from S1 breast epithelial cells, and the relative abundance of each circRNA was measured. Further validation of the subcellular localization of the circRNAs was performed by fractionation and measuring the levels of the circRNAs using GAPDH as a cytoplasmic control and *MALAT1* and U6 as nuclear controls, which were enriched in the respective cellular compartments (Figure 1F). circ_753 was mainly enriched in the nuclear fraction, while circ_755 was predominantly detected in the cytoplasmic fraction (Figure 1F). The nuclear localization was ∼70% for circ_753, and the cytoplasmic localization was ∼70% for circ_755. This compartmental difference was statistically significant and indicates that, although both circRNAs originate from the same Cx43 host transcript, they are not distributed in the same way inside the cell.

### 2.2. Down-Regulation of Exon-Intron circ_753 Induces Higher Expression Levels of miR-182, Implicated in Breast Cancer Progression, than Exonic circ_755

**Figure 2:**
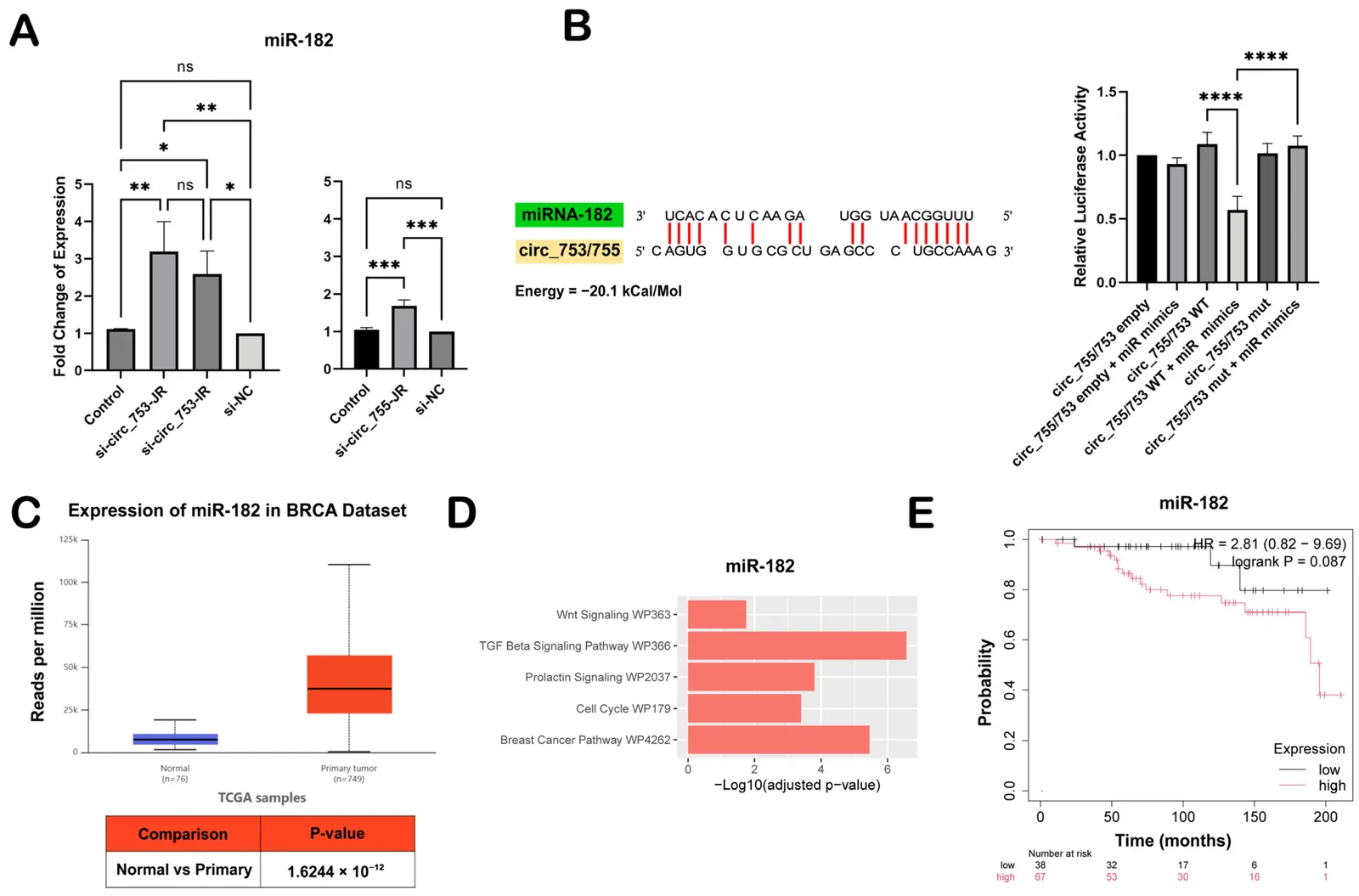
miR-182-5p rises after depletion of circ_753 and circ_755, interacts with their shared back-splice sequence, and is elevated in TCGA breast tumors with cancer-relevant pathway enrichment. (**A**) RT-qPCR of miR-182-5p (24 h). Bar graphs show relative expression as fold change (2^−ΔΔCt^) with *SNORD44* as the small-RNA endogenous control, and si-NC set to 1.0. Left: circ_753 experiment set (Control, si-circ_753-JR, si-circ_753-IR, si-NC). Right: circ_755 experiment set (Control, si-circ_755-JR, si-NC). Bars = mean ± SEM; *n* = 3; each measured in technical triplicate. (**B**) Binding schematic and dual-luciferase assay. Left: sequence pairing shows seed complementarity and minimum-free-energy hybridization between miR-182-5p and the shared circ_753/circ_755 sequence. Right: reporter assay in S1 cells; bars show relative luciferase activity (Firefly/Renilla, scaled to empty + no mimic = 1.0). Conditions: empty vector (±miR-182 mimic), circ_753/755-WT (±mimic), and circ_753/755-mut (±mimic). Statistical comparisons shown are circ_753/755-WT versus circ_753/755-WT + miR-182 mimic, and circ_753/755-WT + miR-182 mimic versus circ_753/755-mut + miR-182 mimic. Bars = mean ± SEM, *n* = 3. (**C**) TCGA-BRCA-Box-and-whisker plot of hsa-miR-182 in normal (*n* = 76) versus primary tumor (*n* = 749) samples. The two-group comparison *p*-value is indicated beneath the plot. (**D**) Pathway enrichment for miR-182 targets. Bar chart of selected pathways ranked by −log10(adjusted *p*) from enrichment analysis of high-confidence miR-182-5p target genes; (**E**) Survival stratification. Kaplan–Meier overall-survival curves for high versus low miR-182 expression (cohort split as indicated by the plotting tool). The hazard ratio (HR) with 95% CI and log-rank *p*-value appear on the plot; numbers-at-risk are shown below the x-axis. Reagents and nomenclature used throughout. JR = junction region siRNA directed to the circRNA back-splice junction; IR = intronic region siRNA unique to circ_753; si-NC = non-targeting negative-control siRNA; miR mimic = synthetic miR-182-5p mimic. Significance notation: ns, not significant (*p* ≥ 0.05); * *p* < 0.05; ** *p* < 0.01; *** *p* < 0.001; **** *p* < 0.0001.

We evaluated the expression of miR-182 after knockdown of *GJA1*-derived circRNAs to investigate whether they regulate oncogenic miRNA activity. When circ_753 or circ_755 was transiently silenced in S1 breast epithelial cells, miR-182 expression increased by 3-fold and 1.5-fold, respectively; thus, knockdown of circ_753 alone induced a more noticeable increase in miR-182 expression (Figure 2A). Next, we examined whether circ_755 and circ_753 have the potential to interact with miR-182 directly. The predicted miR-182-responsive sequence showed sequence-dependent responsiveness to miR-182 in the reporter assay (Figure 2B). TCGA breast cancer data analysis verified that primary tumors have significantly higher levels of miR-182 expression than normal tissue (*p* = 1.62 × 10^−12^) (Figure 2C). Pathway enrichment analysis, conducted to further establish the relevance of miR-182 in breast cancer, showed strong associations with signaling pathways related to cancer, such as Wnt, TGF-β, cell cycle, and specifically the breast cancer pathway (Figure 2D). Finally, a survival analysis using breast cancer patient datasets showed a non-significant trend toward lower overall survival probability in patients with higher miR-182 expression (HR = 2.81, 95% CI: 0.82–9.69; log-rank *p* = 0.087) (Figure 2E). Although this trend is consistent with the tumor-promoting role of miR-182, the association did not reach statistical significance and therefore needs to be confirmed in larger and independent patient cohorts. Collectively, these findings support an inverse relationship between the expression of circ_755 and circ_753 and miR-182 levels, together with an association of miR-182 up-regulation with tumor-promoting pathways in BC.

### 2.3. Stable Knockdown of circ_753, Independent of the Parental Gene, Induces More Pronounced Disruption of Polarity and Lumen Integrity, and Enhances Invasiveness Compared to the Stable Knockdown of circ_755 in 3D Culture of Breast Epithelial Cells

**Figure 3:**
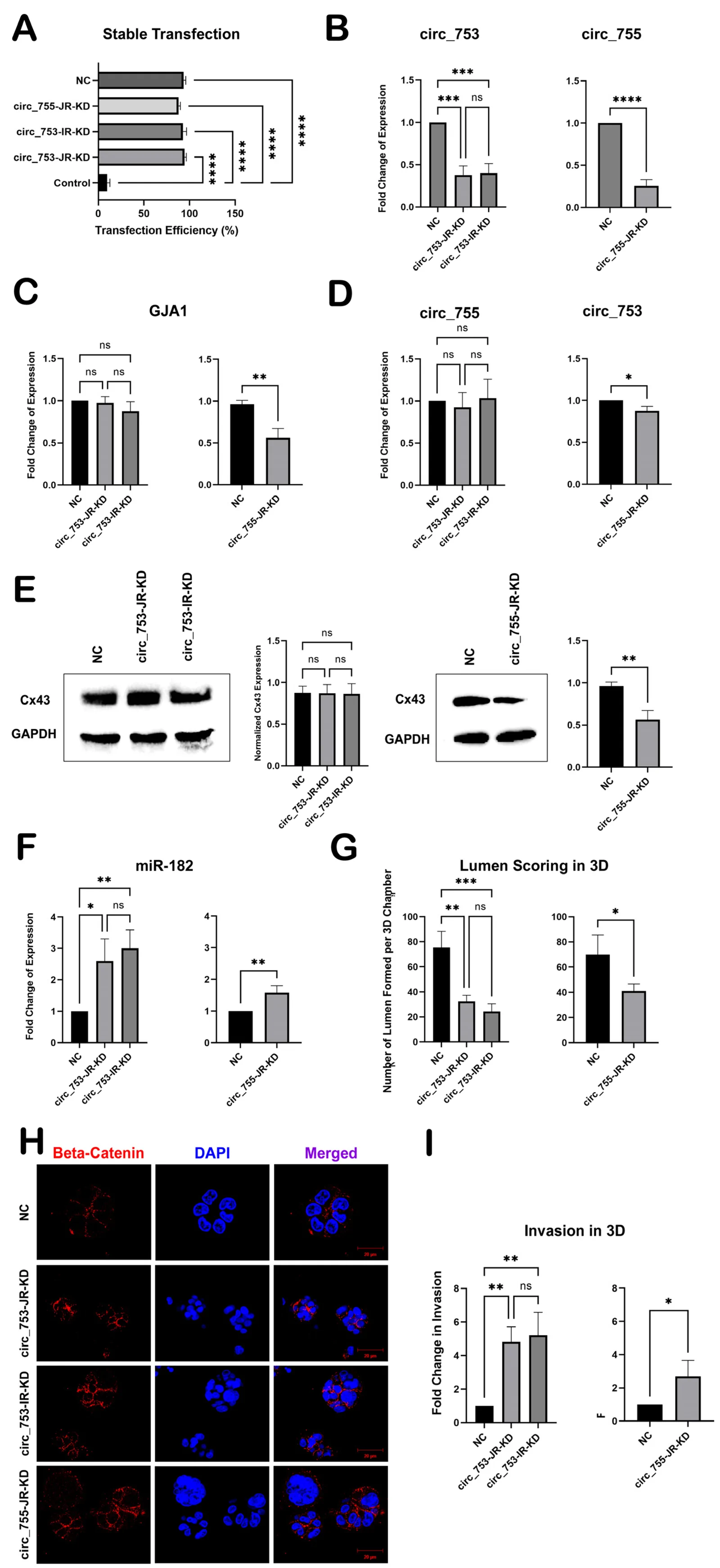
Stable knockdown of *GJA1*-derived circRNAs in S1 acini: circ_753 depletion most strongly disrupts polarity and lumen formation and enhances invasion compared with circ_755 depletion. (**A**) Efficiency of lentiviral stable transduction. The bar graph summarizes transduction efficiency as the percentage of GFP-positive cells within the analysis gate for Control, NC, circ_753-JR-KD, circ_753-IR-KD, and circ_755-JR-KD conditions. Bars represent mean ± SEM (*n* = 3 biologically independent experiments). (**B**) RT-qPCR assessment of stable circRNA knockdown efficiency. Bar graphs display relative expression on the y-axis as fold change (2^−ΔΔCt^), with 18S as the internal control and NC set to 1.0. Left: circ_753 expression in NC, circ_753-JR-KD, and circ_753-IR-KD cells. Right: circ_755 expression in NC and circ_755-JR-KD cells. Bars represent mean ± SEM (*n* = 3 biologically independent experiments). (**C**) RT-qPCR assessment of *GJA1* mRNA expression following stable circRNA knockdown. Left: *GJA1* expression in NC, circ_753-JR-KD, and circ_753-IR-KD cells. Right: *GJA1* expression in NC and circ_755-JR-KD cells. Relative expression is presented as fold change (2^−ΔΔCt^), with GAPDH as the internal control and NC set to 1.0. Bars represent mean ± SEM (*n* = 3 biologically independent experiments). (**D**) RT-qPCR assessment of cross-isoform effects following stable circRNA knockdown. Left: circ_755 expression following circ_753 knockdown with circ_753-JR-KD or circ_753-IR-KD. Right: circ_753 expression following circ_755 knockdown with circ_755-JR-KD. Relative expression is presented as fold change (2^−ΔΔCt^), with 18S as the internal control and NC set to 1.0. Bars represent the mean ± SEM (*n* = 3 biologically independent experiments). (**E**) Cx43 protein expression by immunoblot. Left: representative Western blots for Cx43, with GAPDH as the loading control, in NC, circ_753-JR-KD, and circ_753-IR-KD cells, together with densitometric quantification of Cx43 expression normalized to GAPDH. Right: representative Western blots and corresponding densitometric quantification of Cx43 expression in NC and circ_755-JR-KD cells. Bars represent the mean ± SEM (*n* = 3 biologically independent experiments). (**F**) RT-qPCR analysis of miR-182-5p expression following stable circRNA knockdown. Left: miR-182-5p expression in NC, circ_753-JR-KD, and circ_753-IR-KD cells. Right: miR-182-5p expression in NC and circ_755-JR-KD cells. Relative expression was calculated using the 2^−ΔΔCt^ method with *SNORD44* as the internal control, and NC set to 1.0. Bars represent mean ± SEM (*n* = 3 biologically independent experiments). (**G**) Lumen formation in 3D culture. Bar graphs show the number of lumens formed per 3D chamber, determined by blinded counting. Left: NC, circ_753-JR-KD, and circ_753-IR-KD cells. Right: NC and circ_755-JR-KD cells. Bars represent mean ± SEM (n ≥ 3 chambers per condition). (**H**) β-catenin immunofluorescence in 3D culture. Representative confocal images show β-catenin (red), DAPI (blue), and merged channels for NC, circ_753-JR-KD, circ_753-IR-KD, and circ_755-JR-KD acini. Images are representative of 3 independent experiments; scale bars = 20 μm. (**I**) Invasion in 3D culture. Bar graphs show fold change in invasion relative to NC = 1.0. Left: NC, circ_753-JR-KD, and circ_753-IR-KD cells. Right: NC and circ_755-JR-KD cells. Bars represent mean ± SEM (*n* = 3 biologically independent experiments). Controls and nomenclature used throughout: Control = untreated/untransduced cells; NC = non-targeting control vector; JR-KD = stable lentiviral knockdown directed against the circRNA back-splice junction; IR-KD = stable lentiviral knockdown directed against the intronic sequence unique to circ_753. Significance notation: ns, not significant (*p* ≥ 0.05); * *p* < 0.05; ** *p* < 0.01; *** *p* < 0.001; **** *p* < 0.0001.

Using the lentiviral system, we established stable knockdowns of circ_755 and circ_753 in S1 breast epithelial cells, thereby creating a 3D culture model that allowed us to assess polarity and perform immunofluorescence analysis in S1 cells after 8–12 days in culture. Effective transduction was validated through cell sorting analysis, detecting GFP-positive cells (Figure 3A and Figure S1C,D). RT-qPCR validation showed that the targeted circRNAs were specifically down-regulated, with circ_753 expression reduced to approximately 0.4-fold of the control level using both circ_753-JR and circ_753-IR knockdown, and circ_755 expression reduced to approximately 0.25-fold of the control level (Figure 3B). While circ_753 knockdown had no discernible effect on the parental gene, circ_755 knockdown decreased Cx43 mRNA and protein expression, with expression reduced to approximately 0.6-fold of control levels, which is consistent with the results of transient silencing (Figure 3C,E). Further cross-silencing analysis showed that circ_753 knockdown did not significantly affect circ_755 expression, whereas circ_755 knockdown resulted in a small but significant reduction in circ_753 expression (Figure 3D). Higher levels of miR-182 were associated with both knockdowns, with a 2.5-fold and 3-fold increase in circ_753-JR and circ_753-IR, respectively, and a 1.5-fold increase in circ_755-JR (Figure 3F), similar to the effects following the transient transfection. Next, we examined the effect on polarity of the breast epithelial cells in 3D culture models, as a marker for early tumorigenic events. Cellular polarity, as indicated by immunofluorescence staining of β-catenin, was disrupted in circRNA-silenced cells compared to controls, with more pronounced disruption in circ_753 knockdown cultures (Figure 3H). Quantitative analysis of acini revealed a marked reduction in lumen formation following circ_753 knockdown, with approximately 25–30 lumen-containing structures compared with approximately 75 in control cultures, while circ_755 knockdown resulted in approximately 40 lumen-containing structures compared with approximately 70 in controls (Figure 3G). Lastly, invasion assays demonstrated that loss of either circ_753 or circ_755 enhanced the ability of breast epithelial cells to invade, with a greater increase in circ_753-KD cells compared to circ_755-KD cells (Figure 3I). These results collectively demonstrate that the silencing of circRNAs derived from Cx43 results in the acquisition of tumor-initiating phenotypes, with circ_753 having a more pronounced effect on the ability to invade and epithelial polarity.

### 2.4. Stable Downregulation of Intron-Exonic circ_753 Yields Distinct Changes in miR-182 Target Genes and in Other Candidate miR-182-Sponging circRNAs

**Figure 4:**
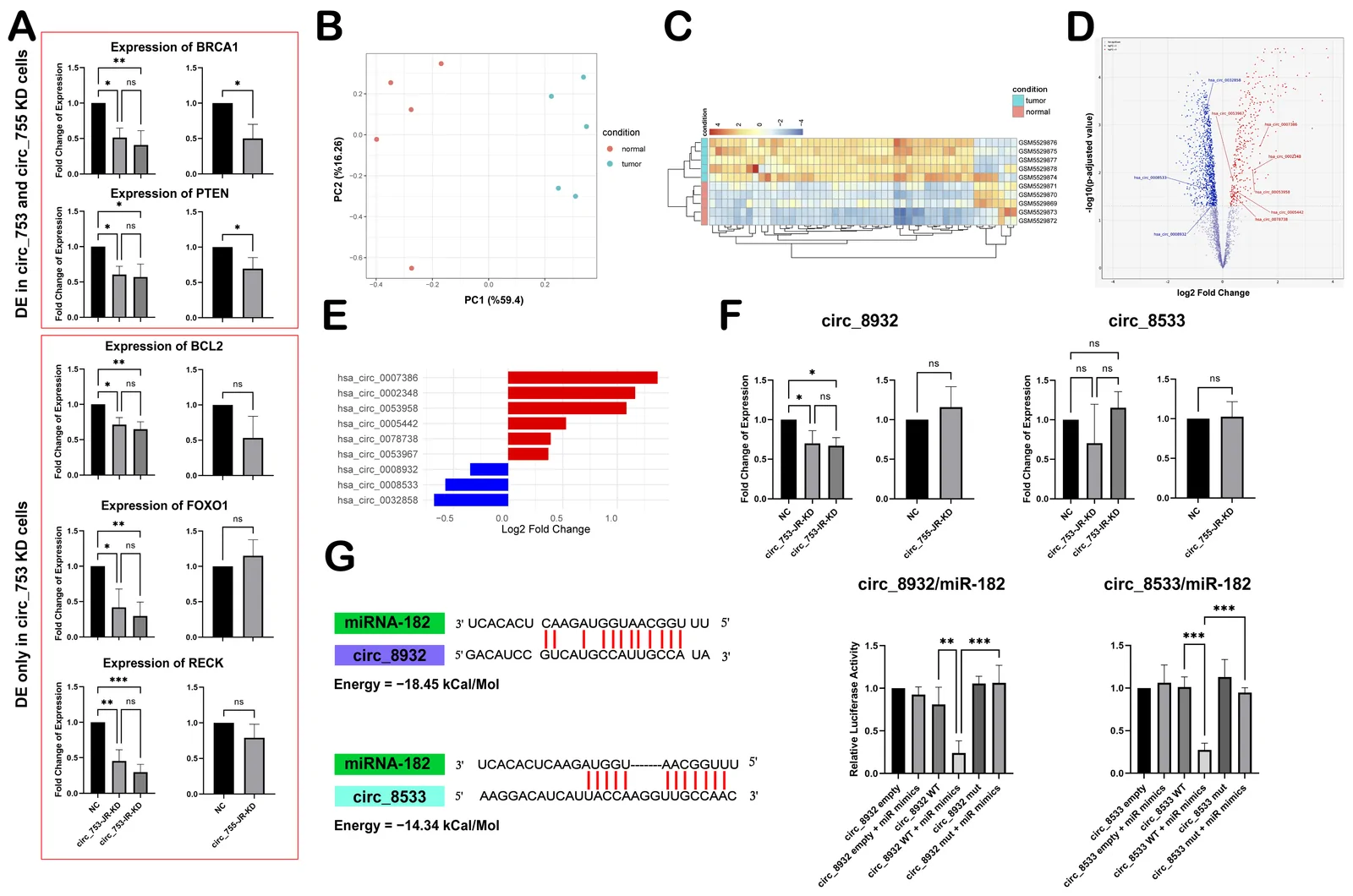
Stable circ_753 knockdown alters tumor-suppressor gene expression and reveals additional miR-182-interacting circRNAs, circ_0008932 (circ_8932) and circ_0008533 (circ_8533), through public dataset analysis and experimental validation. (**A**) RT-qPCR analysis of candidate downstream target genes following stable circRNA knockdown. Bar graphs display relative expression on the y-axis as fold change (2^−ΔΔCt^), with GAPDH as the internal control and NC set to 1.0. The upper group (“DE in circ_753 and circ_755 KD cells”) shows *BRCA1* and *PTEN* expression, whereas the lower group (“DE only in circ_753 KD cells”) shows *BCL2*, *FOXO1*, and *RECK* expression. For each transcript, the left graph corresponds to the circ_753 knockdown set (NC, circ_753-JR-KD, circ_753-IR-KD), and the right graph corresponds to the circ_755 knockdown set (NC, circ_755-JR-KD). Bars represent mean ± SEM (*n* = 3 biologically independent experiments). (**B**) Principal component analysis (PCA) of circRNA expression profiles from the public dataset GSE182471. Samples are colored according to condition (normal versus tumor), demonstrating separation of the two groups based on their circRNA expression profiles. (**C**) Heat map of differentially expressed circRNAs identified from GSE182471, with hierarchical clustering of samples and circRNAs. (**D**) Volcano plot of differential circRNA expression between tumor and normal samples in GSE182471. The x-axis represents log_2 fold change and the y-axis represents −log_10 adjusted *p* value; selected differentially expressed circRNAs are highlighted. (**E**) Ranking of selected differentially expressed circRNAs according to log_2 fold change. Red bars indicate circRNAs with higher expression in tumor samples, whereas blue bars indicate circRNAs with lower expression in tumor samples. hsa_circ_8932 and hsa_circ_8533 were selected for subsequent experimental validation. (**F**) RT-qPCR validation of circ_8932 and circ_8533 following stable circRNA knockdown. Bar graphs display relative expression on the y-axis as fold change (2^−ΔΔCt^), with 18S as the internal control and NC set to 1.0. For circ_8932 (left) and circ_8533 (right), the left graph corresponds to the circ_753 knockdown set (NC, circ_753-JR-KD, circ_753-IR-KD), and the right graph corresponds to the circ_755 knockdown set (NC, circ_755-JR-KD). Bars represent mean ± SEM (*n* = 3 biologically independent experiments). (**G**) Predicted miR-182-5p binding and dual-luciferase reporter validation of circ_8932 and circ_8533. Left: sequence-pairing diagrams showing the predicted interaction of miR-182-5p with circ_8932 and circ_8533; predicted minimum free energy values are indicated. Right: dual-luciferase reporter assays evaluating the interaction of miR-182-5p with the predicted binding sites in circ_8932 and circ_8533. Bar graphs show relative luciferase activity (Firefly/Renilla), normalized to the empty-vector control without miR-182-5p mimic = 1.0. Conditions include empty vector, WT reporter containing the predicted miR-182-5p binding site, and mutant reporter containing a disrupted binding site, each in the absence or presence of miR-182-5p mimic. Statistical comparisons were performed between WT and WT + miR-182-5p mimic to assess miR-182-mediated repression of the WT reporter, and between WT + miR-182-5p mimic and MUT + miR-182-5p mimic to assess the specificity of this response following mutation of the predicted binding site. Bars represent mean ± SEM (*n* = 3 biologically independent experiments). Controls and nomenclature used throughout: NC = non-targeting control vector; KD = stable lentiviral knockdown; JR-KD = stable knockdown directed against the circRNA back-splice junction; IR-KD = stable knockdown directed against the intronic sequence unique to circ_753; WT = wild-type reporter; mut = mutant reporter; DE = differentially expressed; mimic = synthetic miR-182-5p mimic. Significance notation: ns, not significant (*p* ≥ 0.05); * *p* < 0.05; ** *p* < 0.01; *** *p* < 0.001.

To investigate the molecular pathways involved in the phenotypic differences between knockdown of circ_755 and circ_753, we looked at the mRNA levels of a number of downstream target genes regulated by miR-182. The mRNA levels of *BRCA1* and *PTEN* were decreased by ~0.5-fold in circ_755-KD and circ_753-KD cells. The mRNA levels of *BCL2*, *FOXO1*, and *RECK* were downregulated only in circ_753 knockdown cells, with respective decreases of 0.4-, 0.6-, and 0.6-fold. Still, their expression was unaffected by circ_755 knockdown (Figure 4A). To further investigate whether the reduction in *FOXO1* and *RECK* following circ_753 depletion was related to the increase in miR-182-5p, a molecular rescue experiment was performed using a miR-182-5p inhibitor. Since *FOXO1* and *RECK* were significantly downregulated following circ_753 knockdown in the initial experiments, the analysis included NC, circ_753-JR-KD, and circ_753-IR-KD cells treated with either a control inhibitor or a miR-182-5p inhibitor. Effective inhibition of miR-182-5p was confirmed in all conditions (Figure S2A). Importantly, direct comparison of each circ_753-KD condition treated with the control inhibitor versus the corresponding condition treated with the miR-182-5p inhibitor showed a significant increase in *FOXO1* expression in both circ_753-JR-KD and circ_753-IR-KD cells following miR-182-5p inhibition (Figure S2B). Similarly, *RECK* expression significantly increased following miR-182-5p inhibition in both circ_753 knockdown conditions (Figure S2C). These findings indicate that inhibition of miR-182-5p partially reverses the repression of *FOXO1* and *RECK* associated with circ_753 depletion, supporting a functional contribution of miR-182-5p to the regulation of these downstream targets. To account for the more pronounced rise in miR-182 after circ_753 knockdown, compared with circ_755, we investigated whether loss of circ_753 coincides with reduced levels of additional circRNAs that interact with miR-182. Bioinformatic analysis of GEO datasets (accession number: GSE182471) was used to identify additional circRNAs proposed to interact with miR-182. Based on circRNA profiles, principal component analysis (PCA) and hierarchical clustering showed a distinct separation of tumor and normal samples (Figure 4B,C). Several circRNAs were found to be significantly dysregulated; circ_8932 and hsa_circ_0008533 (circ_8533) were predicted to interact with miR-182 with an opposite expression pattern (Figure 4D,E). Validation in S1 cells demonstrated that circ_753 knockdown decreased the expression of circ_8932, which is in line with the increased levels of miR-182 observed. The expression of circ_8533, also proposed to interact with miR-182, remained unchanged (Figure 4F). Lastly, the predicted miR-182-responsive sequences within circ_8533 and circ_8932 were evaluated using dual-luciferase reporter assays. Both reporter constructs showed sequence-dependent responsiveness to miR-182, as demonstrated by reduced luciferase activity following co-transfection with miR-182 mimics, while mutation of the predicted binding site abolished this response (Figure 4G). These results collectively indicate the presence of a reciprocal circRNA/miR-182 regulatory network, with significant implications in breast tumorigenesis. Circ_753 has a broader regulatory impact than circ_755, as its loss results in a more pronounced upregulation of miR-182 and a downregulation of several tumor-suppressive genes, including *BCL2*, *FOXO1*, and *RECK*. The molecular rescue experiment further supports a functional contribution of miR-182-5p to the regulation of *FOXO1* and *RECK*, as miR-182-5p inhibition significantly increased the expression of both genes in circ_753-JR-KD and circ_753-IR-KD cells compared with their corresponding control-inhibitor conditions (Figure S2). Furthermore, mature miR-182-5p abundance was increased following circ_753 knockdown, which was also associated with decreased expression of another circRNA predicted to interact with miR-182, circ_8932. The more pronounced molecular changes observed following circ_753 silencing were accompanied by broader perturbation of downstream target genes (Figure 4A) and decreased expression of additional circRNAs predicted to interact with miR-182. Although circ_753 silencing was also associated with increased invasion, lumen disruption, and β-catenin mislocalization, the present rescue experiment was limited to the molecular effects on *FOXO1* and *RECK* and does not establish that miR-182-5p mediates these phenotypic changes.

### 2.5. EV-Associated circRNA and miR-182 Profiles Reflect Intracellular Changes Following circ_753 and circ_755 Knockdown in Breast Epithelial Cells

**Figure 5:**
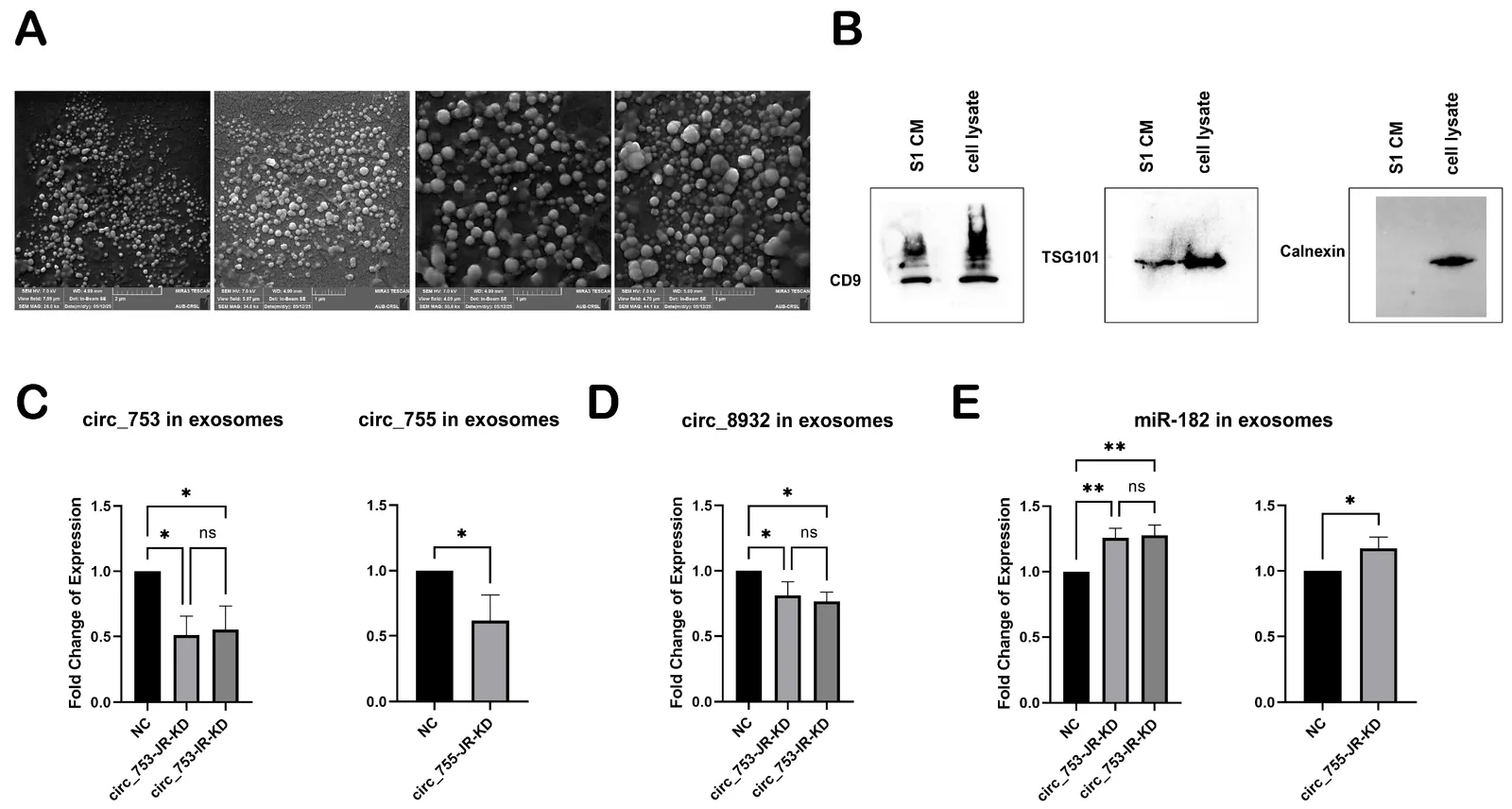
Characterization and RNA profiling of extracellular vesicles released by S1 breast epithelial cells. (**A**) Scanning electron microscopy. Representative micrographs of vesicles isolated from S1 conditioned medium (CM), showing rounded particles consistent with EVs. Scale bars as indicated on each panel; acquisition settings are shown in the scanning electron microscopy overlays. (**B**) Exosome markers immunoblot. Western blot for the EV markers CD9 and TSG101, together with the cellular marker Calnexin, in material purified from S1 CM and in cell lysate. CD9 and TSG101 were detected in the S1 CM-derived vesicle fraction, whereas Calnexin was detected in the cell lysate and was absent from the S1 CM fraction, supporting EV enrichment and low cellular contamination. (**C**–**E**) RT-qPCR of exosomal RNA cargo. Bar graphs report relative expression (2^−ΔΔCt^) for the indicated RNAs in RNA extracted from the vesicle fraction. For circRNAs (circ_753, circ_755, hsa_circ_8932), values are normalized to the designated exosomal reference control and scaled relative to NC = 1.0; for miR-182-5p, values are normalized to a small-RNA reference and scaled relative to NC = 1.0 (see Methods for reference details). Conditions: NC (non-targeting control vector), circ_753-JR-KD, circ_753-IR-KD, and circ_755-JR-KD (where shown). Bars = mean ± SEM; *n* = 3. Nomenclature. NC = non-targeting control vector; JR-KD = junction-region stable lentiviral knockdown targeting the circRNA back-splice junction; IR-KD = intronic-region stable knockdown unique to circ_753. Significance notation: ns, not significant (*p* ≥ 0.05); * *p* < 0.05; ** *p* < 0.01.

Extracellular vesicles (EVs) can carry regulatory RNAs that reflect the molecular state of their cells of origin. Both circRNAs and miRNAs can be incorporated into EVs and protected from extracellular degradation [16,17,18]. In breast cancer, EV-associated noncoding RNAs, including miR-182-5p and circRNAs, have been implicated in intercellular communication and disease-associated phenotypes [16,17,18]. We therefore examined whether the circRNA/miR-182 changes observed following circ_753 or circ_755 depletion were also detectable in EVs released by S1 breast epithelial cells. Accordingly, EVs isolated from the conditioned medium of circ_755-KD and circ_753-KD S1 breast epithelial cells were assayed to determine whether *GJA1*-derived circRNAs and miR-182 were detectable in the vesicle-associated RNA fraction and whether their expression patterns reflected the corresponding intracellular changes. Vesicle-like structures with sizes consistent with EVs were confirmed by scanning electron microscopy (Figure 5A). EV enrichment was further supported by Western blot analysis showing CD9 and TSG101 in the isolated vesicle fraction, together with the absence of Calnexin, an endoplasmic reticulum marker, supporting the purity of the EV preparation (Figure 5B). Next, we examined whether EVs contain circ_755 and circ_753, and if so, whether their expression levels correspond to the knockdown trend. In fact, circ_753 silencing decreased circ_753 expression in the purified vesicles by 0.5-fold, and circ_755 knockdown decreased circ_755 expression by 0.4-fold (Figure 5C). Furthermore, circ_753 knockdown resulted in a 0.3-fold decrease in circ_8932 expression, which is thought to interact with miR-182, highlighting the impact of circ_753 loss on the expression of circ_8932 in EVs (Figure 5D). Importantly, miRNA analysis showed that EVs from both circ_755- and circ_753-silenced cells had around a 1.3-fold increase in the levels of miR-182 expression (Figure 5E).

### 2.6. Aligned Loss of circRNAs and Their Parental Genes Correlates with miR-182 Up-Regulation in Tumorigenic Breast Epithelial T4-2 Cells Compared to Non-Neoplastic Breast Epithelial S1 Cells in 3D Culture

**Figure 6:**
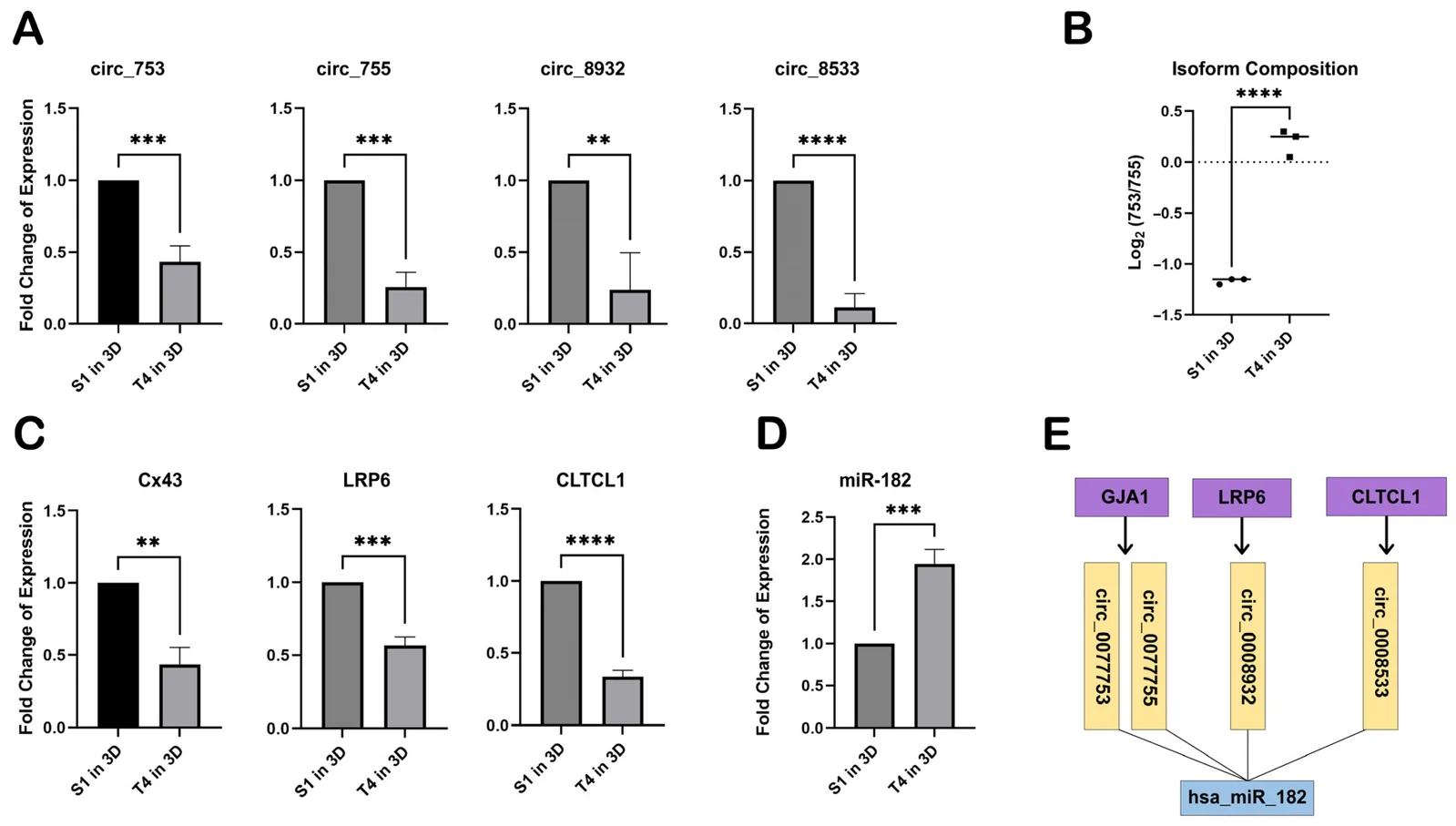
S1 vs. T4-2 in 3D: circRNAs and host mRNAs decline, miR-182 rises, and isoform composition shifts toward circ_753. (**A**) circRNA abundance in 3D acini. Bar graphs show relative expression (2^−ΔΔCt^) for circ_753, circ_755, hsa_circ_8932, and hsa_circ_8533, each 18S-normalized and expressed relative to S1 in 3D = 1.0. Groups: S1 in 3D and T4-2 in 3D. Bars = mean ± SEM (*n* = 3). (**B**) Isoform composition within condition. Scatter/mean plot of log_2_(753/755) calculated from 2^−ΔCt^ values (each isoform normalized to GAPDH within the same sample). The dotted line at 0 marks equal composition. Negative values indicate circ_755 dominance; positive values indicate circ_753 dominance. (**C**) Host-gene mRNA expression. *GJA1*/Cx43, *LRP6*, and *CLTCL1* mRNAs quantified by 2^−ΔΔCt^, GAPDH-normalized, relative to S1 in 3D = 1.0. Bars = mean ± SEM, *n* = 3 per group. (**D**) miR-182-5p in 3D acini. 2^−ΔΔCt^ values *SNORD44*-normalized, relative to S1 in 3D = 1.0 (y-axis: fold change). Bars = mean ± SEM, *n* = 3 per group. (**E**) Schematic summary. Host genes (Cx43, *LRP6*, *CLTCL1*) and their circRNAs (circ_753, circ_755, circ_8932, circ_8533) depicted as converging inputs to hsa-miR-182, illustrating the measured layers in panels A-D. Nomenclature. S1 = non-tumorigenic human breast epithelial cells; T4-2 = tumorigenic counterpart; circ_753/circ_755 = Cx43-derived circRNAs (EIciRNA and exonic, respectively). Significance notation: ns, not significant (*p* ≥ 0.05); ** *p* < 0.01; *** *p* < 0.001; **** *p* < 0.0001.

We compared the expression of the circRNAs of interest, along with their parental genes and the oncogenic miR-182, in tumorigenic T4-2 versus non-tumorigenic S1 breast epithelial cells to determine whether the circRNA/miR-182 network is altered in tumorigenic cells. When cultured in 3D, S1 cells undergo acinar morphogenesis and acquire polarity over 8 days, and over the same period, T4-2 cells form tumor nodules with irregular shapes, deprived of polarity [19]. CircRNAs predicted to interact with miR-182, namely circ_755, circ_753, circ_8932, and circ_8533, were found to be significantly down-regulated in T4-2 cells, as compared to S1 cells cultured in 3D, with a 0.6-, 0.7-, 0.7-, and 0.9-fold change, respectively (Figure 6A). Isoform-abundance analysis of *GJA1*-derived circRNAs revealed a compositional shift within the Cx43-derived circRNA pool. While overall circRNA levels decline in T4-2 cells, circ_755 is disproportionately reduced relative to circ_753, yielding a lower circ_755: circ_753 ratio in the tumorigenic state. This isoform rebalancing points to altered back-splicing in T4-2 cells and, together with the global circRNA loss, is consistent with altered regulation of oncogenic miR-182 by circRNAs predicted to interact with it (Figure 6B). The parental genes of the selected circRNAs, *GJA1*, *LRP6*, and *CLTCL1*, were all significantly downregulated in T4-2 cells compared to S1 cells cultured in 3D, with 0.6-, 0.5-, and 0.6-fold changes (Figure 6C). Simultaneously, T4-2 cells exhibited a 1-fold increase in miR-182 expression, alongside reduced levels of circRNAs predicted to interact with miR-182 (Figure 6D). The schematic model (Figure 6E) illustrates the parental genes, *GJA1*, *LRP6*, and *CLTCL1*, which are down-regulated in T4-2 cells compared to S1 cells cultured in 3D, together with their corresponding circRNAs (circ_755, circ_753, circ_8932, and circ_8533). These circRNAs follow the expression pattern of their parental genes and are predicted to interact with miR-182. This network links the loss of several circRNAs and their parental genes with the abnormal up-regulation of oncogenic miR-182, highlighting a broader axis of Cx43-derived circRNA/miR-182 dysregulation in T4-2 tumorigenic cells.

## 3. Discussion

Our data reveal that two distinct circRNAs from the *GJA1* locus have nonredundant roles in mammary epithelial homeostasis. Circ_755, an entirely exonic circRNA, and circ_753, an EIciRNA, share an identical predicted miR-182-responsive sequence (Figure 1A and Figure 2B). A gene can generate multiple circRNAs that do not replicate each other’s roles. For instance, the β-catenin (*CTNNB1*) gene yields one ecircRNA that enhances proliferation by stabilizing the β-catenin through a translated peptide [20], and another intronic circRNA from the same locus that drives pathway activation via a transcriptional scaffold [21], two differential routes to oncogenic pressure. Likewise, the titin (*TTN*) gene produces one *TTN* circRNA crucial for orchestrating muscle-specific splicing programs by creating a back-splice motif essential for *SRSF10*/*RBM20*-dependent splicing of key muscle genes [22], and another intronic circRNA has been shown to recruit *PURB*-containing repressive complexes at the *TTN* locus, dampening host-gene transcription and, as a consequence, blunting myogenic differentiation [23]. Further, in the heart, the *Slc8a1* (NCX1) locus illustrates isoform-specific circRNA biology. Exonic heart-related circRNA (HRCR) sequesters miR-223 and protects against hypertrophy and heart failure [24]. Conversely, circ*Slc8a1* (exon 2), the most abundant cardiac circRNA, plays distinct roles, including miR-133 binding under hypertrophic stress and mitochondrial support of ATP production, whose loss or silencing causes cardiac dysfunction [25]. These are not simple opposites of HRCR; they underscore that circRNAs from the same gene execute nonredundant programs. Together, these precedents make a simple point: circRNAs derived from the “same gene” do not exert the “same work”. In that context, our data are consistent with distinct functional behavior of these *GJA1*-derived circRNAs in the mammary epithelium: circ_753 and circ_755, both derived from *GJA1*, appear to contribute along a similar trend but to different extents to early tumor-initiating behavior in human breast epithelial cells, reinforcing the view that the gene origin alone cannot predict circRNA function and that each isoform warrants separate mechanistic and phenotypic interrogation. We mapped circ_755 and confirmed that it is fully exonic and embedded within the *GJA1*/Cx43 mRNA sequence (Figure 1A). Critically, the back-splice junction, usually the anchor for isoform-specific silencing, contains a 16-nt stretch ideally shared with Cx43 mRNA and present in circ_753. This precludes a junction-directed knockdown of circ_755 alone without risking collateral *GJA1* mRNA and circ_753 suppression, which was shown in our data (Figure 1 and Figure 3). Accordingly, the molecular and phenotypic effects observed following circ_755 knockdown cannot be attributed exclusively to circ_755 depletion, as the concomitant reduction in *GJA1*/Cx43 expression may also contribute to these effects. This distinction is important when comparing circ_755 and circ_753 knockdown, since circ_753 could be targeted independently of the parental *GJA1* transcript, whereas circ_755 could not. Mapping the sequence of circ_753, we found that it has an additional 46-nt intronic sequence. Given this exon–intron architecture, we also examined whether circ_753 and circ_755 differ in their nuclear/cytoplasmic distribution. Our fractionation data showed that circ_753 was mainly enriched in the nuclear fraction, whereas circ_755 was predominantly detected in the cytoplasmic fraction (Figure 1F). This difference fits with the intron-containing nature of circ_753 and the fully exonic structure of circ_755. These two *GJA1*-derived circRNAs differ not only in sequence composition and knockdown behavior but also in their cellular compartmentalization.

Importantly, miR-182 is a well-established oncomiR in breast cancer and other malignancies, with a broad network of mRNA targets involved in cell survival, proliferation, and invasiveness [26]. Silencing circ_753 and circ_755 each led to significant downregulation of *BRCA1* and *PTEN* (Figure 4A). However, circ_753 knockdown, which was accompanied by a more pronounced increase in miR-182-5p, additionally resulted in significant downregulation of several tumor suppressor genes, including *FOXO1* and *RECK*, whereas circ_755 knockdown produced a more modest effect. This pattern is consistent with the established regulatory functions of miR-182 in cancer. For example, *FOXO1*, a key pro-apoptotic transcription factor, is a well-documented direct target of miR-182 [26,27], and its repression has been linked to enhanced cell survival in breast cancer. The reduction in *BCL2* may reflect indirect regulation or context-dependent miR-182 activity; It is worth noting that some studies also indicate that miR-182 can directly bind and inhibit *BCL2* in specific cell types [28]; however, in breast epithelial models its effects on apoptotic signaling may also occur indirectly through *FOXO1*-associated pathways [26,27]. Another critical target of miR-182 affected by the circ_753 KD is *RECK*, Reversion-inducing Cysteine-rich Protein with Kazal motifs, a membrane-anchored inhibitor of matrix metalloproteinases and an established metastasis suppressor [27,29,30,31,32]. Reduced *RECK* expression upon circ_753 KD is in line with published data showing that miR-182 activation downstream of β-catenin signaling in breast cancer drives invasiveness by directly targeting and down-regulating *RECK* [31]. Loss of *RECK* would promote extracellular matrix degradation and invasion, linking the circRNA/miR-182 axis to early invasive capabilities [31]. Collectively, the concomitant repression of *FOXO1* and *RECK* illustrates how increased miR-182 activity could simultaneously affect multiple tumor-suppressive pathways involving cell survival and invasion. This functional association was further supported by the miR-182-5p inhibition experiment. Since *FOXO1* and *RECK* were significantly reduced specifically following circ_753 depletion, their expression was examined after miR-182-5p inhibition in circ_753-JR-KD and circ_753-IR-KD cells. In both conditions, *FOXO1* and *RECK* were no longer significantly different from the NC condition following miR-182-5p inhibition (Figure S2). These findings support the contribution of increased miR-182-5p to the downstream molecular effects associated with circ_753 depletion. However, the present rescue experiment was limited to molecular targets and does not establish that the observed lumen-formation or invasive phenotypes are directly mediated through miR-182-5p. *BRCA1* and *PTEN* also showed decreases following circRNA depletion; notably, miR-182 has previously been shown to suppress *BRCA1* expression and impair DNA damage responses, providing a possible explanation for the observed reduction in *BRCA1* [27]. Overall, the stronger repression of several miR-182-associated genes following circ_753 knockdown parallels the more pronounced increase in mature miR-182-5p and supports a functional connection between circ_753 and the oncogenic miR-182 regulatory network. The attenuation of *RECK* and *FOXO1* downregulation following miR-182 inhibition further strengthens this functional association and suggests that at least part of the downstream effect of circ_753 depletion is mediated through miR-182 (Figure S2).

Importantly, however, the mechanism through which circ_753 depletion increases mature miR-182-5p remains unresolved. This distinction is particularly relevant given the predominant nuclear localization of circ_753. Although circRNAs can regulate miRNA activity through a classical ceRNA mechanism, this model is most readily applicable to cytoplasmic circRNAs that sequester miRNAs and are present at sufficient abundance relative to their cognate miRNAs and competing targets [33,34,35,36]. In contrast, nuclear-enriched circRNAs, particularly exon–intron circRNAs, can exert distinct regulatory functions, including modulation of transcription through interactions with components of the nuclear transcriptional machinery [37]. Moreover, mature miRNA abundance can be regulated at several levels, including primary miRNA transcription, Drosha- and Dicer-mediated processing, Argonaute loading, stabilization, and degradation [38]. Furthermore, CircRNA–miRNA interactions may also influence miRNA stability and turnover rather than functioning solely through stoichiometric sequestration, as shown for the CDR1as/ciRS-7–miR-7 regulatory network [39,40]. Therefore, the increased abundance of mature miR-182-5p following circ_753 knockdown may result from alterations in miR-182 transcription, processing, stability, or functional availability rather than, or in addition to, a classical cytoplasmic ceRNA mechanism.

Functionally, both circRNA knockdowns disrupted epithelial morphology in 3D culture, but to different extents. Loss of either circRNA caused loss of acinar polarity (as seen by mislocalized β-catenin), failure of lumen formation, and increased invasive capacity; in every assay, the phenotype was more pronounced for circ_753 KD (Figure 3). Notably, this was observed even though circ_755 knockdown was accompanied by partial reductions in both circ_753 and *GJA1*/Cx43 expression. However, the phenotypic effects observed following circ_755 knockdown should be interpreted with caution, as they may reflect the combined consequences of circ_755 depletion and Cx43 loss rather than a circ_755-specific effect alone. These phenotypes are hallmarks of premalignant transformation: for instance, *GJA1*-null or *GJA1*-silenced S1 acini similarly lose polarity and form multilayered structures [15]. Thus, circ_753 appears critical for maintaining acinar architecture, even independent of Cx43 loss. Moreover, the Wnt/β-catenin pathway was enriched in our computational analysis of miR-182 targets, and studies have shown that β-catenin upregulates miR-182 in aggressive breast cancer cells [27]. The altered β-catenin staining that we observed upon circRNA knockdown aligns with our previous work [41], which suggests that Cx43 loss, and here, circRNA loss, feed back onto Wnt signaling, further driving invasive capability.

Bioinformatic analysis of breast cancer circRNA datasets highlighted two circRNAs: circ_8932 (from the *LRP6* gene, a co-receptor in the Wnt/β-catenin pathway), linking miR-182 regulation to Wnt signaling components, and circ_8533 (from *CLTCL1*, a clathrin heavy chain gene), which were down-regulated in tumor samples and predicted to bind miR-182 (Figure 4). We confirmed in S1 cells that circ_8932 is down-regulated when circ_753 is silenced, whereas circ_8533 is unchanged. Crucially, luciferase assays demonstrated that both circ_8932 and circ_8533 bind miR-182 directly; wild-type reporter activity was suppressed by miR-182, whereas mutant reporters were not. Indeed, in an independent context, circ_8932 has been reported to sponge miR-182 in esophageal squamous carcinoma cells, thereby upregulating the oncogene Myc and promoting tumor cell proliferation and invasion [42]. These findings expand the potential miR-182 competing endogenous RNA (ceRNA) network, suggesting that multiple circRNAs from different genomic loci may contribute to the regulation of the same oncomiR. Similar networks of overlapping miRNA sponging have been described in cancer, underscoring the concept of competitive endogenous RNA regulation [43]. The parent genes of these circRNAs, *LRP6* and *CLTCL1*, were also downregulated in T4-2 tumor cells, suggesting that tumor cells may coordinately suppress both host gene expression and circRNA production (Figure 6). This coordinated loss would relieve miR-182 inhibition on all fronts, amplifying oncogenic signaling. Such redundancy may help explain why loss of one circRNA can perturb multiple pathways: miR-182 targets extend across apoptosis, DNA repair, and motility (e.g., *FOXO1*, *RECK*, *BRCA1*), so disrupting the circRNA network unleashes a broad tumor-promoting program. This broadens the scope of the circRNA/miR-182 axis beyond *GJA1* alone, indicating a network of gene-circRNA/miR interactions operative in breast cancer initiation. It will be important for future studies to map this network comprehensively and determine whether these circRNAs function redundantly or have distinct subsets of miR-182 targets. Nonetheless, our current data support a potential regulatory role for miR-182 in early oncogenic signaling and suggest that circRNAs derived from tumor suppressor genes, including *GJA1*, *LRP6*, and *CLTCL1*, may collectively modulate miR-182-associated pathways.

EVs are increasingly recognized as carriers of regulatory RNAs and mediators of intercellular communication, including in BC. EV-associated circRNAs and miRNAs are protected from degradation in the extracellular environment and transferred to recipient cells. For example, circPSMA1 associated with EVs derived from breast cancer has been reported to promote proliferative and migratory phenotypes through the miR-637/*AKT1*/β-catenin axis [16], and EV-associated miR-182-5p has been shown to promote endothelial-cell migration and angiogenic signaling through the *CMTM7*/*EGFR*/AKT pathway [17]. More recently, TNBC-derived EV-associated circRHCG was reported to influence macrophage polarization and metastatic progression [18]. These observations provided the rationale for investigating whether the intracellular circRNA/miR-182 alterations identified in our S1 model were also reflected in released EVs. In the present study, scanning electron microscopy revealed rounded vesicular structures with morphological features consistent with EVs, while Western blot analysis demonstrated enrichment of the established EV-associated proteins CD9 and TSG101 in the isolated vesicle fraction (Figure 5A,B). We found that both *GJA1*-derived circRNAs were detectable inside exosome-like vesicles released by breast epithelial cells. This observation is consistent with the emerging recognition that circRNAs are selectively and abundantly packaged into EVs [44]. EcircRNAs are often enriched in exosomal fractions, likely because their cytoplasmic location makes them accessible for sorting into budding vesicles [45]. The exonic vs. exon–intronic nature distinction of these two circRNAs matters: ecircRNAs are typically cytoplasmic and often act as miRNA sponges [33,46], while EIciRNAs tend to accumulate in the nucleus and modulate transcriptional programs [37,47,48]. In keeping with the architecture-to-function logic, the cytoplasmic localization of the exonic circ_755 is more compatible with miRNA-related regulation; however, the dual-luciferase assay only demonstrates functional responsiveness of the shared predicted miR-182-binding sequence and does not establish endogenous miR-182 sponging. In contrast, its exon–intron counterpart, circ_753, might contribute to additional nucleus-proximal activities, regulating the expression of a variety of downstream genes. Given its exon–intron architecture and based on the nuclear/cytoplasmic fractionation data (Figure 1F), circ_753 was mainly enriched in the nuclear compartment, whereas the fully exonic circ_755 was predominantly detected in the cytoplasmic fraction. We posit that circ_753 may act as a nuclear scaffold, beyond canonical miRNA sponging, potentially engaging U1/RNA polymerase II-linked complexes to fine-tune transcription, a behavior described for other intron-containing circRNAs [47]. Silencing each circRNA in breast epithelial cells revealed a similar trend but to different extents, with phenotypic and molecular consequences, underscoring their functional divergence despite a common origin from *GJA1*-hnRNA [37,47,48,49]. The fact that circ_755 was readily found in the exosome compartment, whereas the intron-containing circ_753 was less so, is in alignment with their proposed cellular localizations. Such packaging is biologically meaningful: exosomal circRNAs are now recognized as stable carriers of regulatory RNAs that facilitate cell–cell communication in cancer [43].

Finally, we note that our knockdown-induced network changes correspond to inherent differences between normal and malignant breast epithelia. The T4-2 cells, which form nodules in 3D, displayed the same signature as that induced by circRNA knockdown: down-regulation of *GJA1*-derived circRNAs, circ_753 and circ_755, loss of circ_8932/8533, and up-regulation of miR-182, compared to nontumorigenic S1 cells. This observation reinforces the pathophysiological relevance of our findings. It also aligns with published data: miR-182 is frequently overexpressed in primary breast tumors and is even detectable at higher levels in patient serum compared to controls [27], with a non-significant trend toward poorer survival in the present analysis (Figure 2E). Conversely, *GJA1* expression [41], in addition to *CLTCL1* [50] and *LRP6* expression, tends to be down-regulated in aggressive breast cancer, as we observed in T4-2 cells.

According to multiple studies [51,52,53,54], the same gene can produce several circRNA isoforms, and which one is more abundant often shifts in a stage-dependent context. Cancer vs. normal tissues, and even different disease stages, show isoforms switching, where a tumor favors a different circular transcript than its healthy counterpart. The abundance of readouts aligns with this logic: junction-directed silencing of both circRNAs drives a larger decrease in circ_755 expression compared to circ_753, and RT-qPCR Cq values place circ_755 earlier than circ_753, indicating higher baseline abundance. Consistently, when we profiled T4-2 (tumorigenic) versus S1 (nontumorigenic) cells, circ_755 expression decreased more strongly relative to circ_753, suggesting isoform remodeling rather than a uniform loss of both *GJA1*-derived circRNAs. Circ_755 appears predominant at baseline, whereas the tumorigenic T4-2 state shows a disproportionate reduction in circ_755 relative to circ_753, resulting in a compositional shift toward circ_753 (Figure 6B).

Although the findings in this manuscript should be interpreted in the context of certain limitations, they nevertheless provide insight into how circRNA isoforms derived from the same parent gene may contribute differently to early breast cancer initiation events. Worth noting is the unique intronic sequence of circ_753, which enabled the selective silencing of circ_753 without affecting its parental *GJA1* transcript. In contrast, the sequence overlap between circ_755 and the linear *GJA1* transcript limited the ability to achieve selective silencing of circ_755 independently of the limited downregulation of its parental *GJA1* transcript. Accordingly, direct comparisons between circ_753 and circ_755 knockdown should be interpreted with caution, since the two perturbations are not experimentally equivalent: circ_753 depletion is independent of *GJA1*, whereas circ_755 depletion is accompanied by partial *GJA1*/Cx43 suppression. Rescue experiments using an shRNA-resistant circ_755 construct, together with comparison to a miR-182-binding-site mutant, would help further distinguish circ_755-specific effects from those resulting from collateral *GJA1* suppression. In addition, although the dual-luciferase assay supports the functional responsiveness of the predicted shared miR-182-binding sequence, it does not directly demonstrate an endogenous interaction between miR-182 and the circRNAs. Additional approaches, such as AGO2-RNA immunoprecipitation or biotinylated miR-182 pulldown, would provide stronger evidence of this interaction. Therefore, the precise mechanism by which circ_753 regulates the abundance and/or availability of miR-182 remains to be determined. Lastly, most experiments were performed in non-neoplastic S1 breast epithelial cells. While this model is useful for studying changes associated with breast cancer initiation events, and some experiments were performed in a 3D culture system to better recapitulate epithelial architecture and morphogenesis, it does not fully reflect the complexity of the breast tissue microenvironment.

## 4. Materials and Methods

### 4.1. CircRNAs Sequence Characterization

The sequence of selected circRNAs was obtained from Circular RNA Interactome (https://circinteractome.nia.nih.gov/index.html, accessed on 15 March 2024), managed by the National Institutes of Health (NIH), and circBase (https://www.circbase.org/, accessed on 15 October 2024). Sequences of each circRNA were then mapped using the Ensembl genome browser (https://asia.ensembl.org/index.html, accessed on 20 June 2024) to identify exonic and intronic regions. The sequence of each circRNA was also aligned against the sequence of its respective parental gene. All siRNAs and primers designed were checked for specificity using the NCBI BLAST web-based sequence alignment tool (https://blast.ncbi.nlm.nih.gov/BlastAlign.cgi, accessed on 20 November 2024) hosted by the National Library of Medicine (NLM).

### 4.2. Cell Culture

The nontumorigenic ER-negative HMT-3522 S1 (S1) human mammary epithelial cells, with passages ranging from 60 to 65, were cultured as a monolayer on plastic (2D culture) in serum-free H14 medium DMEM-F12 medium (GIBCO BRL, St. Louis, MO, USA), containing 250 ng/mL insulin (Boehringer Mannheim, Indianapolis, IN, USA), 10 μg/mL transferrin (Sigma, St Louis, MO, USA), 2.6 ng/mL sodium selenite (BD Biosciences, San Jose, CA, USA), 10-10 M estradiol (Sigma), 1.4 μM hydrocortisone (BD Biosciences), 5 μg/mL ovine prolactin (NIDDK-oPRL-21 (AFP-10692C), National Hormone & Peptide Program), and 10 ng/mL epidermal growth factor (EGF; BD Biosciences) at a temperature of 37 °C and with 5% CO_2_ in a humidified incubator [55,56,57]. The same medium was used for HMT-3522 T4-2 (T4-2) except with no EGF added. The H14 medium was replaced every 2–3 days. For 2D cultures, cells were plated on plastic substrate at a density of 2.3 × 10^4^ cells/cm^2^. T4-2 cells were cultured on plastic coated with Purecoll. The drip method of 3D culture was used to induce the formation of acini. Briefly, cells were plated on MatrigelTM (50 μL/cm^2^; Corning, 354234, New York, NY, USA) at a density of 4.2 × 10^4^ cells/cm^2^ in the presence of culture medium containing 5% MatrigelTM. To achieve acinar differentiation (typically achieved on day 8 or 9), EGF was omitted from the culture medium on day 7 [56,58].

### 4.3. Cell Lines, Authentication, and Mycoplasma Testing

HMT-3522 S1 cells (RRID: CVCL_2499) were obtained in 2000 from Mina J. Bissell’s laboratory (USA) [59]. STR profiling has not been performed recently in France; therefore, cell identity is routinely monitored by phenotypic validation in 3D culture (morphogenesis/phenotype consistency), with additional genetic/STR testing performed when required. Mycoplasma contamination was assessed using the MycoAlert detection kit (Lonza, Basel, Switzerland, LT07-318) on 4 October 2024, and the test was negative (L2/L1 ratios ~0.56–0.62 across tested S1 samples).

HMT-3522 T4-2 cells (RRID: CVCL_2501) were obtained in 2000 from Mina J. Bissell’s laboratory (USA) [59]. STR profiling has not been performed recently in France; therefore, cell identity is routinely monitored by phenotypic validation in 3D culture (morphogenesis/phenotype consistency), with additional genetic/STR testing performed when required. Mycoplasma contamination was assessed using the MycoAlert detection kit (Lonza, LT07-318) on 26 April 2024, and the test was negative (L2/L1 ratio ~0.63 for the tested T4-2 sample).

### 4.4. Transient Transfection

S1 cells were seeded in 6-well or 12-well tissue culture plates. When cells reached 60–70% confluency, typically around day 5, complete growth medium was replaced. After two h, cells were transfected with FAM-labeled siRNA against circ_753 or circ_755 or a sham (NC) sequence, which were obtained from GenePharma (Shanghai, China). The sense and antisense sequences (5′–3′) of all siRNAs used in these experiments are listed in Table 1. For every well of a 6-well plate, 1.5 μL of the target siRNA was diluted in 300 μL of Opti-MEM in a microcentrifuge tube and incubated for 5 min at room temperature. In another microcentrifuge tube, 9 μL of Lipofectamine RNAiMAX (Thermo Fisher Scientific, Waltham, MA, USA) Transfection Reagent was diluted in 300 μL of Opti-MEM. The diluted siRNA and the diluted Lipofectamine were mixed and incubated for 10 min at room temperature. Then, 250 μL of the mixture was added to the corresponding wells. Volumes were proportionally adjusted for a 12-well plate or a 4-well chambered slide (used for 3D cell culture). Cells used for RNA extraction were collected 24 h post-transfection. We monitored transfection efficiency using a fluorescent control siRNA simultaneously with the Guava EasyCyte™ machine (Luminex Corporation, Austin, TX, USA) and then verified it using RT-qPCR with primers for each circRNA.

### 4.5. Stable Transfection Through Lentiviral Infection

The 3rd generation lentiviral packaging mix by ABM was utilized to generate lentiviral particles expressing shRNA to down-regulate circ_753 or circ_755, and another particle set as a negative control with GFP label and puromycin resistance. The shRNA sequences (5′–3′) cloned into the LV3(H1/GFP & Puro) lentiviral expression vectors are listed in Table 2. Briefly, 10 μg of shRNA-GFP-tagged expression vector (GenePharma, Shanghai, China) for circ_753 or circ_755 or the negative control vector was mixed with 10 μg of third-generation packaging mix (ABM, Vancouver, Canada), then diluted in 1 mL of serum-free, antibiotic-free medium for each 10 cm dish. The mixture was transfected into the amphoteric 293T packaging cells using Invitrogen™ Lentifectin™ Transfection reagent (Thermo Fisher Scientific, Waltham, MA, USA) according to the manufacturer’s protocol (80 μL) and incubated at 37 °C for 5–8 h; then, 0.65 mL FBS was added to the 10 cm dish and incubated overnight at 37 °C. The transfection medium was replaced with complete medium and incubated overnight at 37 °C. The first viral harvest in the supernatant was collected at 3000 rpm for 15 min at 4 °C, filtered, and stored at 4 °C for the next day. Complete medium was added again to the cells, and a second harvest was collected and filtered the next day and added to the first harvest that was kept at 4 °C. The pooled harvest was aliquoted and stored at −80 °C until use. The viral titer of the first harvest was approximately 106 IU/mL. As shown in our previous studies, the puromycin drug selection killing curve showed that a concentration of 0.5 μg/mL of puromycin was optimal for selecting the infected cells as per the manufacturer’s protocol (ABM, Vancouver, BC, Canada) [15]. Multiplicity of infection (MOI) was calculated as Product Titer (IU/mL) × Virus Volume (mL)/Total Cell Number, and 3 MOI was used for the S1 cells, as recommended by ABM for breast cell lines. For infection, filtered lentiviral supernatants were applied to monolayers of S1 cells on day 4 in culture. Cells were incubated with hexadimethrine bromide (Polybrene; 8 μg/mL; Sigma, St. Louis, MO, USA) for 8 h. The infection medium was removed, and cells were incubated in regular H14 medium for 24 h. Infection was repeated two additional times on days 5 and 6 of culture, and selection with puromycin dihydrochloride (0.5 µg/mL; Gibco™, USA, A-1113803) was started 72 h after the last infection (day 9). shRNA-infected cells against circ_753 or circ_755, and control-infected cells, were maintained, propagated, and plated similarly to S1 cells, but the H14 medium was supplemented with puromycin for selection. The stability of silencing was regularly assessed in different cell passages with fluorescence microscopy.

### 4.6. Guava Flow Cytometry

Cells were seeded in 2D in a 12-well plate. At 60–70% confluency, cells were either left untransfected or transiently transfected using FAM-labeled siRNA or sham (GenePharma, Shanghai, China). After 24 h of transfection, cells were trypsinized and resuspended in 1× PBS. For flow cytometry, the Guava EasyCyte™ System was used with the laser CFSE (carboxyfluorescein) of maximum emission 517 nm to determine the fluorescence of each sample. In each sample, 10,000 nongated events were acquired and consequently analyzed using GuavaSoft™ 2.7 Software.

### 4.7. RNA Extraction and Quantification

TRI Reagent (T9424, Sigma) was used for RNA extraction according to the manufacturer’s protocol. To extract RNA from cells in a well in a 6-well plate, 300 μL of TRI reagent was added to the culture plate and scraped with a scraper. The lysate was then passed through a 1000 μL tip several times and transferred to a microcentrifuge tube. Samples were left to sit for 5 min at room temperature. Sixty μL of chloroform was added, and the samples were vortexed for 15 s. Samples were left to sit for 10 min at room temperature, then centrifuged at 12,000× *g* for 15 min at 2–8 °C. The aqueous phase was transferred to a fresh tube. Then, 150 μL of isopropanol was added, and the samples were vortexed for 2 s. The samples were left to sit for 10 min at room temperature, then centrifuged at 12,000× *g* for 10 min at 2–8 °C. The supernatant was aspirated, and the RNA pellet was washed with 300 μL of 75% ethanol. Samples were vortexed for 15 s, then centrifuged at 7500× *g* for 5 min at 2–8 °C. The ethanol was removed, and the wash was repeated once more. Centrifugation steps during ethanol washes were performed at 12,000× *g* if the pellet floated upon vortexing. Ethanol was removed, and the pellet was left to dry at room temperature by opening the cap for 20–45 min. Next, 100 μL of diethyl pyrocarbonate (DEPC) water was added to each tube without resuspending the pellet. Samples were incubated at 55 °C for 15 min using a dry heater. The pellet was resuspended on ice, and samples were stored properly. RNA quality was assessed by measuring A260/230 and A260/280 on a NanoDrop. 260/280 values above 1.8 and 260/230 values around two were considered acceptable.

### 4.8. Validation of circRNA Amplification

Divergent primers were designed to amplify the predicted back-splice junctions of circ_753 and circ_755. Primer sequences and predicted amplicons were evaluated in silico using the NCBI BLAST web-based sequence alignment tool (https://blast.ncbi.nlm.nih.gov/BlastAlign.cgi, accessed on 20 November 2024) hosted by the National Library of Medicine (NLM). to assess specificity and potential nonspecific amplification from unrelated genomic or transcript sequences. PCR products were resolved by agarose gel electrophoresis to confirm the presence of discrete products at the expected molecular sizes, and no-template controls were included to monitor for contamination or nonspecific amplification. The circular nature of circ_753 and circ_755 was further assessed by RNase R treatment followed by RT-qPCR, using the linear transcripts *GJA1* and GAPDH as controls. During initial assay optimization, RNase R-treated and untreated samples showed comparable Cq values and melt-curve profiles for circ_753 (Cq 23 and 21, respectively) and circ_755 (Cq 22 and 20, respectively), whereas the linear controls were markedly depleted following RNase R treatment, with *GJA1* shifting from Cq 18 to Cq 38 and GAPDH from Cq 16 to undetectable. Therefore, subsequent RT-qPCR analyses of circ_753 and circ_755 were performed using untreated total RNA.

### 4.9. RT-qPCR for circRNA

cDNA synthesis was performed using the Quantitect reverse transcription kit (Qiagen cat, Hilden, Germany, # 205311) for 1 µg of total RNA as per the manufacturer’s protocol. However, instead of random primers, specific primers for circ_753 and circ_755 and the endogenous control 18S were utilized in the reverse transcription reaction. After the addition of the RNA samples to the reverse-transcription mix, the reaction (25 μL per reaction) was incubated at 25 °C for 10 min, then at 50 °C for 30 min, then at 85 °C for 5 min. Then, qPCR was performed in a 20 μL reaction volume consisting of the following: 4 μL of diluted cDNA, 4 μL of primers (total forward and reverse of 5 μM concentration of each primer), 2 μL of DEPC water, and 10 μL of SYBR Green JumpStart Taq ReadyMixTM (Sigma, S4438). Each sample was performed in duplicate for each target. One master mix was prepared for each primer, and qPCRs were run separately for different targets. Primer sequences (5′–3′) and corresponding amplicon lengths for all targets are provided in Table 3. The RT-qPCR was performed using a Bio-Rad CFX96 Real-Time System, C1000 Thermal Cycler (Feldkirchen, Germany). The reaction was run at 95 °C for 5 min, 40 cycles of amplification at 95 °C for 15 s, annealing, and extension at 58.5 °C for 1 min. Data were normalized using the ΔΔCT method. At least three independent biological replicates were used for each experiment, and each condition had three technical replicates.

### 4.10. RT-qPCR for mRNA

cDNA synthesis was performed using the Quantitect reverse transcription kit (Qiagen cat # 205311) for 1 μg of total RNA as per the manufacturer’s protocol with random primers. The 20 μL reaction mix was incubated at 42 °C for 15 min, then at 95 °C for 3 min. After the reverse transcription was complete, RT-qPCR was run, as previously described, using a Bio-Rad CFX96 Real-Time System and a C1000 Thermal Cycler (Feldkirchen, Germany). Primer sequences (5′–3′) and corresponding amplicon lengths for all targets are provided in Table 3. The reaction was run for 95 °C for 2 min, 40 cycles of amplification at 95 °C for 15 s, 56 °C for 1: 30 s, 72 °C for 1 min, and a final elongation step of 72 °C for 10 min. Data were normalized using the ΔΔCT method. At least three independent biological replicates were used for each experiment, and each condition had three technical replicates.

### 4.11. RT-qPCR for miRNAs

Reverse transcription of 10 ng of the total RNA was performed using the microRNA Reverse Transcription Kit (Applied Biosystems, Waltham, MA, USA) according to the manufacturer’s instructions and as previously described by Nassar et al. [15]. Briefly, small nuclear RNA *SNORD44* and miR-182 primers were purchased as part of the microRNA Assays Kit (Applied Biosystems, Waltham, MA, USA) with validated efficiency. cDNA synthesis was carried out for miR-182 in each reaction with the endogenous control, *SNORD44*. RT-qPCR was performed using a Bio-Rad CFX96 Real-Time System and a C1000 Thermal Cycler (Feldkirchen, Germany). RT-qPCR was performed in 10 μL-reactions consisting of: 5 μL of SYBR Green JumpStart Taq ReadyMix (SIGMA S4438), 1 μL of the corresponding microRNA primer set, 1 μL of DEPC water, and 3 μL of cDNA. Each sample was performed in duplicate for each target. The cycling conditions were 95 °C for 2 min and 40 cycles of 95 °C for 10 s and an annealing and extension step of 56 °C for 1 min. Data were normalized using the ΔΔCT method. At least three independent biological replicates were used for each experiment, and each condition had three technical replicates.

### 4.12. miR-182-5p Inhibition and Molecular Rescue Analysis

To determine whether the depletion of circ_753 leads to the reduction in *FOXO1* and *RECK* through miR-182-5p, circ_753-JR-KD and circ_753-IR-KD S1 cells were transfected with a miR-182-5p inhibitor from Gene Pharma (Shanghai, China) at a final concentration of 25pmol using Lipofectamine 3000, Thermo Fisher Scientific, according to the manufacturer’s instructions. Total RNA was isolated, and miR-182-5p expression was assessed by RT-qPCR to confirm successful inhibition after 24 h of inhibitor treatment. Following that, *FOXO1* and *RECK* expression were evaluated by RT-qPCR as previously described. These targets were selected for the rescue analysis because both were significantly downregulated following circ_753 depletion in the original experiments, whereas neither showed significant downregulation following circ_755 depletion.

### 4.13. Subcellular Fractionation

S1 cells were resuspended in 100 µL of hypotonic buffer A (10mM HEPES, pH 7.5, 0.5 mM DTT, 10 mM KCl, 1.5 mM MgCl_2_ · 6H_2_O, protease inhibitor) and incubated on ice for 10 min before being centrifuged at 1000× *g* for 10 min at 4 °C. Both the supernatant and the pellet were collected for subsequent isolation of the cytoplasmic and nuclear fractions, respectively. To obtain the cytoplasmic fraction, the supernatant was centrifuged at 15,000× *g* for 10 min at 4 °C, and the supernatant was retrieved. To obtain the nuclear fraction, the pellet was washed twice with hypotonic buffer A then resuspended in hypotonic buffer B (10 mM HEPES, pH 7.5, 0.5 mM DTT, 10 mM KCl, 1.5 mM MgCl_2_·6H_2_O, 0.5% Nonidet P-40, and protease inhibitor) and incubated for 30 min at 4 °C with gentle rotation. The sample was then centrifuged at 6000× *g* for 10 min at 4 °C. The pellet was washed again with hypotonic buffer A, then resuspended in RIPA buffer (50 mM Tris-HCl, pH 7.5, 150 mM KCl, 1% Nonidet P-40, 0.5% deoxycholic acid, 0.1% SDS, and 1 mM EDTA, pH 8.0) and incubated for another 30 min at 4 °C with gentle rotation. Finally, the solution was centrifuged at 15,000× *g* for 20 min at 4 °C, and the supernatant was collected as the nuclear fraction. From each fraction, RNA was extracted, and RT-qPCR was performed.

### 4.14. Dual-Luciferase Reporter Assays

Dual-luciferase reporter assays (GenePharma, Shanghai, China) were used to investigate the interaction between circ_753, circ_755, circ_8932, circ_8533, and miR-182. In short, the possible miR-182 binding sequences from these circRNAs (with circ_753 and circ_755 sharing the same sequence) were cloned into the pGL3-control vector downstream of the firefly luciferase reporter gene. Simultaneously, mutant constructs with broken miR-182 seed sequences were made as negative controls. The sequence of the circ_755/53-miR-182-MUT construct used to disrupt the miR-182 seed binding site is provided in Table 2. S1 cells were plated in 24-well plates. The next day, they were co-transfected with 0.5 μg of either wild-type or mutant reporter plasmid, along with 50 nM of hsa-miR-182 mimics or negative control mimics (GenePharma), using Lipofectamine 3000 (Thermo Fisher Scientific). The sense and antisense sequences (5′–3′) of the hsa-miR-182-5p mimics used for co-transfection are provided in Table 1. Following the manufacturer’s instructions for the Dual-Luciferase Reporter Assay System, luciferase activity was measured 48 h after transfection on a Varioskan Flash Multimode Reader (Thermo Fisher Scientific). To assess the effectiveness of the transfection, we compared the activity of firefly luciferase with that of Renilla luciferase from the same vector. The data are presented as the relative luciferase activity of cells transfected with control mimics. For each condition, three independent biological replicates were used in triplicate wells for all experiments.

### 4.15. Bioinformatics Analysis

Circular RNA Interactome (https://circinteractome.nia.nih.gov/, accessed on 15 March 2024) and starBase v3.0 (http://starbase.sysu.edu.cn/, accessed on 20 March 2024) were used to find predicted circRNA/miRNA interactions. Downstream miR-182 targets were obtained from TargetScanHuman v7.2 (http://www.targetscan.org, accessed on 20 March 2024), miRTarBase (http://mirtarbase.cuhk.edu.cn/, accessed on 20 March 2024), and miRDB (http://mirdb.org, accessed on 20 March 2024). DAVID v6.8 and KEGG pathway annotations were used to do a functional enrichment analysis of miR-182 targets. The significance level was set at *p* < 0.05 after the Benjamini–Hochberg correction. For expression analyses, publicly accessible circRNA and mRNA microarray datasets were acquired from the Gene Expression Omnibus (GEO), specifically GSE182471. DE circRNAs were obtained by GEO2R. The prcomp and pheatmap R functions were used to do principal component analysis (PCA) and hierarchical clustering. The EnhancedVolcano R package was used to make volcano plots, using R version 4.1.1. The Genomic Data Commons (GDC) portal gave us access to transcriptomic and miRNome data for breast cancer patients from the Cancer Genome Atlas (TCGA-BRCA) project. Normalized expression values were expressed in transcripts per million (TPM). KM-Plotter (http://kmplot.com/analysis/, accessed on 15 March 2024) was used to perform Kaplan–Meier survival analyses and log-rank tests to determine the significance of survival. All bioinformatics analyses were performed utilizing standard pipelines.

### 4.16. Lumen Scoring

Stably transfected cells were cultured in 3D as described. Cells were stained on day 12. Briefly, cells were washed twice with 1× PBS, then fixed with 4% formaldehyde in 1× PBS overnight at 4 °C. The next day, cells were washed with 1× PBS, and nuclei were stained with 1 μg/mL Hoechst 33342 (Molecular Probes, Eugene, OR, USA, H3570) in 1× PBS for 10 min at room temperature. They were washed with 1× PBS twice, then mounted in ProLong^®^ Gold antifade reagent 62 (Invitrogen Molecular Probes, Carlsbad, CA, USA, P36930), allowed to dry overnight, and sealed. The slides were examined with a fluorescence microscope. A minimum of 100 acini were analyzed per group.

### 4.17. Trans-Well Migration Assay

Six-well culture plates were fitted with inserts (8 μm pore size). DMEM-F12 supplemented with 10% FBS was added below the insert. 3 × 105 S1 cells were plated in the inserts in DMEM-F12 supplemented with 1% fetal bovine serum (FBS; Sigma, F-9665). Cells were incubated for 12 h and were then fixed using 4% formaldehyde in 1× PBS for 20 min at room temperature. The cells towards the inside of the insert were removed using a cotton swab, and nuclei of migrating cells at the bottom of the insert were stained with 1 μg/mL Hoechst 33342 (Molecular Probes, H3570) in 1× PBS for 10 min at room temperature. The insert was then cut, mounted on a microscope slide in ProLong^®^ Gold antifade reagent (Invitrogen Molecular Probes, P36930), allowed to dry overnight, and sealed. The inserts were examined with a fluorescence microscope, and the number of migrating cells was counted and reported as a fold change.

### 4.18. Immunofluorescence Staining

S1 cells were plated on 4-well chamber slides to perform 3D culture (FALCON, Mexico City, Mexico, 354104) and were stained by immunofluorescence on day 11 as described earlier [56]. Briefly, cells were washed with 1× PBS and permeabilized with 0.5% peroxide and carbonyl-free Triton X-100 (Amersco, LLC., Framingham, MA, USA, 97062208) in cytoskeleton buffer (100 mM NaCl, 300 mM sucrose, 10 mM PIPES, pH 6.8, 5 mM MgCl_2_, 1 mM pefabloc, 10 μg/mL aprotinin, 250 μM NaF). Cells were washed twice with cytoskeleton buffer and fixed in 4% formaldehyde, after which cells were washed thrice with 50 mM glycine in 1× PBS and blocked with IF 1x containing 10% Goat Serum for 3 h at room temperature. The primary antibody used was mouse monoclonal β-catenin, used at 4 µg/mL (1:50; Santa Cruz Biotechnology, Dallas, TX, USA, sc-7963). Secondary antibodies conjugated with Alexa Fluor 594 (red) (Invitrogen Molecular Probes, Eugene, OR, USA) were used at the manufacturer’s proposed dilutions (1:1000). Nuclei were counterstained with 1 μg/mL Hoechst. Cells were mounted in ProLong^®^ Gold antifade reagent (Thermo Fisher Scientific, P36930), allowed to dry overnight under a coverslip. The slides were then examined and imaged with a laser scanning confocal microscope (Zeiss, Oberkochen, Germany, LSM710). A minimum of 50 acini were analyzed per group.

### 4.19. Preparation of Whole Cell Protein Extracts and Western Blot Analysis

Cells were lysed with a buffer containing protease inhibitors, and proteins were quantified using the DC protein assay (Bio-Rad, Feldkirchen, Germany, 5000116). Equal amounts of protein (50 μg) were separated on 12% polyacrylamide gels, transferred to polyvinylidene difluoride (PVDF) membranes, blocked with 5% low-fat milk in tris-buffered saline with tween 20 (TBST), and immunoblotted with the following primary antibodies: Cx43 (1–2 μg/mL; Santa Cruz Biotechnology, sc-271837, and 5 μg/mL; Invitrogen, 13-8300). Secondary antibodies conjugated to goat anti-mouse (Abcam, Cambridge, UK, ab6787) were used at 0.13 μg/mL. Equal protein loading was verified by immunoblotting for GAPDH (mouse monoclonal, 0.4–1 μg/mL; Santa Cruz Biotechnology, sc-47724). ImageJ software (version 1.54k; National Institutes of Health, Bethesda, MD, USA) was used to measure band intensities. For normalization, GAPDH was used as an internal control. The results were then compared to control conditions.

### 4.20. Exosome Isolation and Characterization

We used EVs from conditioned medium of S1 breast epithelial cells cultured for 3 days in exosome- and serum-free medium (H14 medium, previously described). We collected the conditioned medium and centrifuged it in three steps: first at 300× *g* for 5 min to pellet dead cells, then at 2000× *g* for 20 min to remove cells and debris. After that, we filtered the supernatant with a 0.22 μm filter to remove large EVs. Using an ultracentrifuge at 110,000× *g* for 70 min at 4 °C (T-865 Fixed Angle Rotor for WX+ Ultraspeed Centrifuges, Thermo Fisher Scientific, Waltham, MA, USA), EVs were pelleted, resuspended in 2 mL PBS, pooled together, and then pelleted again under the same conditions. For further analysis, the pellets were stored in 1 mL PBS at −80 °C. For scanning electron microscopy, newly isolated EVs were fixed in 2.5% glutaraldehyde on a coverslip for 20 min at room temperature, washed with PBS, and then dehydrated in a series of graded ethanol solutions. The samples were dried overnight in a desiccator, coated with gold, and imaged with a MIRA3 LMU with an OXFORD EDX detector. For Western blotting, exosomal protein lysates were prepared in RIPA buffer containing protease inhibitors. We used SDS-PAGE to analyze equal amounts of EV-associated proteins and corresponding cell-lysate controls, followed by immunoblotting for the EV-associated markers CD9 and TSG101 and the negative cellular marker Calnexin (ABCAM). The miRNeasy Micro Kit (Qiagen) was used to extract total exosomal RNA for RNA analysis, following the manufacturer’s instructions. We used RT-qPCR to determine the levels of circ_755, circ_753, circ_8932, circ_8533, and miR-182, as explained above (see RT-qPCR and primer design).

### 4.21. Statistical Analysis

At least three independent biological replicates were used for each experiment, and the data were shown as the mean ± standard error of the mean (SEM) unless otherwise noted. Technical triplicates were incorporated for each condition in RT-qPCR, dual-luciferase assays, invasion assays, and exosomal RNA quantifications within each experiment. A two-tailed, unpaired Student’s T-test was used to determine statistical significance between the two groups. A one-way ANOVA followed by the Tukey post hoc test was used to determine statistical significance between more than two groups. A *p*-value of less than 0.05 was deemed statistically significant. We used GraphPad Prism v9.0 (GraphPad Software, San Diego, CA, USA) and R v4.2.0 for all statistical analyses.

## 5. Conclusions

Early breast tumorigenesis is notoriously difficult to detect. Still, our findings raise the possibility that concurrent circRNA downregulation and miR-182 upregulation in EV-associated RNA may warrant further investigation as a potential biomarker signature. This concept echoes prior proposals that a drop in tumor-suppressor gene-derived circRNAs alongside a rise in oncomiRs may be detectable in blood years before a tumor becomes clinically apparent [15]. We need to validate the axis in breast cancer patients, establishing whether the shift precedes histologic change. Parallel in situ assays (Cx43 IHC; circRNAs/miR-182 by RNA ISH) can anchor these signals in tissue architecture. Together, these findings indicate that depletion of circ_755 is accompanied by reduced *GJA1*/Cx43 expression, whereas circ_753 depletion is associated with a more pronounced increase in mature miR-182-5p and broader downstream molecular changes. Loss of either circRNA was associated with tumor initiation-associated phenotypes in S1 acini, although the effects of circ_755 depletion should be interpreted in the context of concomitant *GJA1*/Cx43 reduction. Our findings support an association between *GJA1*-derived circRNAs, miR-182-5p-related molecular changes, and early breast epithelial transformation-associated phenotypes, and show that these RNA alterations are also detectable in released extracellular vesicles.

## Figures and Tables

**Table 1:** siRNA and miRNA mimic sequences. Sense and antisense sequences (5′–3′) of siRNAs targeting circ_755, circ_753 (junction-region and intron-retaining variants), and miR-182 mimics were used for transient transfection assays.

Target	Sense (5′–3′)	Antisense (5′–3′)
circ_755-siRNA	AAACATAGTGGTGCCCAGGCA	UUUGUAUCACCACGGGUCCGU
circ_753-JR-siRNA	ATAAACATAGAGTCAGTGGCT	UAUUUGTAUCUCAGUCACCGA
circ_753-IR-siRNA	AGTGGCTTGAAACTTTTAAAAG	UCACCGAACUUUGAAAAUUUUC
hsa-miR-182-5p mimics	UUUGGCAAUGGUAGAACUCACACU	UGUGAGUUCUACCAUUGCCAAAUU

**Table 2:** shRNA and mutant reporter construct sequences. Sequences of shRNAs cloned into LV3(H1/GFP & Puro) lentiviral expression vectors and of the circ_755/53-miR-182-MUT construct used for knockdown and reporter assays.

Target	Sequence (5′–3′)
circ_755 LV3(H1/GFP&Puro) shRNA Expression Vectors	AAACAUAGUGGUGCCCAGGCATT
circ_753-JR LV3(H1/GFP&Puro) shRNA Expression Vectors	AUAAACAUAGAGUCAGUGGCU
circ_753-IR LV3(H1/GFP&Puro) shRNA Expression Vectors	AGUGGCUUGAAACUUUUAAAAG
circ_755/53-miR-182-MUT	CATGCGACCAGTGGTGCGCTGAGCCCACGGTTTCACTGTGGGTCTCAAAAATATGCTTAT

**Table 3:** Primer sequences used for RT-qPCR. Forward and reverse primer sequences (5′–3′) used for amplification of the indicated genes and circular RNAs, with corresponding amplicon lengths determined using Primer-BLAST.

Target	Forward Primer (5′–3′)	Reverse Primer (5′–3′)	Amplicon Length
*GAPDH*	AAGGTGAAGGTCGGAGTCAAC	GGGGTCATTGATGGCAACAATA	102
*18S*	GTAACCCTTGAACCCCATT	CCATCCAATCGGTAGTAGCG	149
*CRIM1*	CTCTGGCTTATGTGGTTTCC	CGCAGGCTGGCTTTGT	128
*LRP6*	CACTTACTTCCCTGCAATTTTGAACC	TGGCCTGTAGCTGTATGACCTATG	124
*CLTCL1*	TCCTCCCTGTTCGCTTTCAG	CATGTCAATGATCGTGACCTGT	155
*GJA1*	CTTCACTACTTTTAAGCAAAAGAG	TCCCTCCAGCAGTTGAG	106
circ_755	TGTTCAGCTTCATTGCATGT	CCTCCAGCAGTTGAGTAGGC	180
circ_753	TGTTCAGCTTCATTGCATGT	GCCTTCCCCCTAATGAAAGA	194
circ_8932	CCTTGGATTATGATGAAGGCAAA	GAGTGTGTGCTCCAGTCAGT	149
circ_8533	GGATTACAAACCACTGCTCATCA	GCTGTGCGAAGCGGAA	134

## Data Availability

The datasets analyzed during the current study are available in the Gene Expression Omnibus database under accession number: GSE182471 and are available at the following URL: https://www.ncbi.nlm.nih.gov/geo/query/acc.cgi?acc=GSE182471, accessed on 10 December 2023. Breast cancer transcriptomic and miRNA expression data were obtained from The Cancer Genome Atlas Breast Invasive Carcinoma dataset (TCGA-BRCA) through the Genomic Data Commons (GDC) portal. Survival analyses were performed using KM-Plotter. Additional data generated during the current study are available from the corresponding author upon reasonable request.

## Supplementary Materials

The following supporting information can be downloaded at https://www.mdpi.com/article/10.3390/ijms27198849/s1.

## Abbreviations

The following abbreviations are used in this manuscript:

2D	Two-dimensional
3D	Three-dimensional
circRNA	Circular RNA
circ_753	hsa_circ_0077753 (exon–intron/intron-containing isoform)
circ_755	hsa_circ_0077755 (fully exonic isoform)
Cx43	Connexin 43
DMEM	Dulbecco’s Modified Eagle Medium
DMSO	Dimethyl sulfoxide
ECM	Extracellular matrix
EMT	Epithelial–mesenchymal transition
EV(s)	Extracellular vesicle(s)
FBS	Fetal bovine serum
GEO	Gene Expression Omnibus
*GJA1*	Gap junction alpha-1 protein (gene encoding Connexin 43)
IF	Immunofluorescence
miRNA	microRNA
miR-182-5p	microRNA-182-5p
NC	Negative control
PBS	Phosphate-buffered saline
qPCR	Quantitative polymerase chain reaction
RT-qPCR	Reverse transcription quantitative polymerase chain reaction
siRNA	Small interfering RNA
WB	Western blot
