# Supplementary material for: Downregulation of GJA1-Derived circ_753 and circ_755 Differentially Modulates miR-182-5p Expression and Breast Cancer-Associated Phenotypes
Source: Int J Mol Sci. 2026 Oct 3;27(19):8849. doi: 10.3390/ijms27198849 (PMC13664854; doi:10.3390/ijms27198849)
Supplement: Supplementary file 1 [file ijms-27-08849-s001.zip › ijms-4473013-supplementary.pdf]

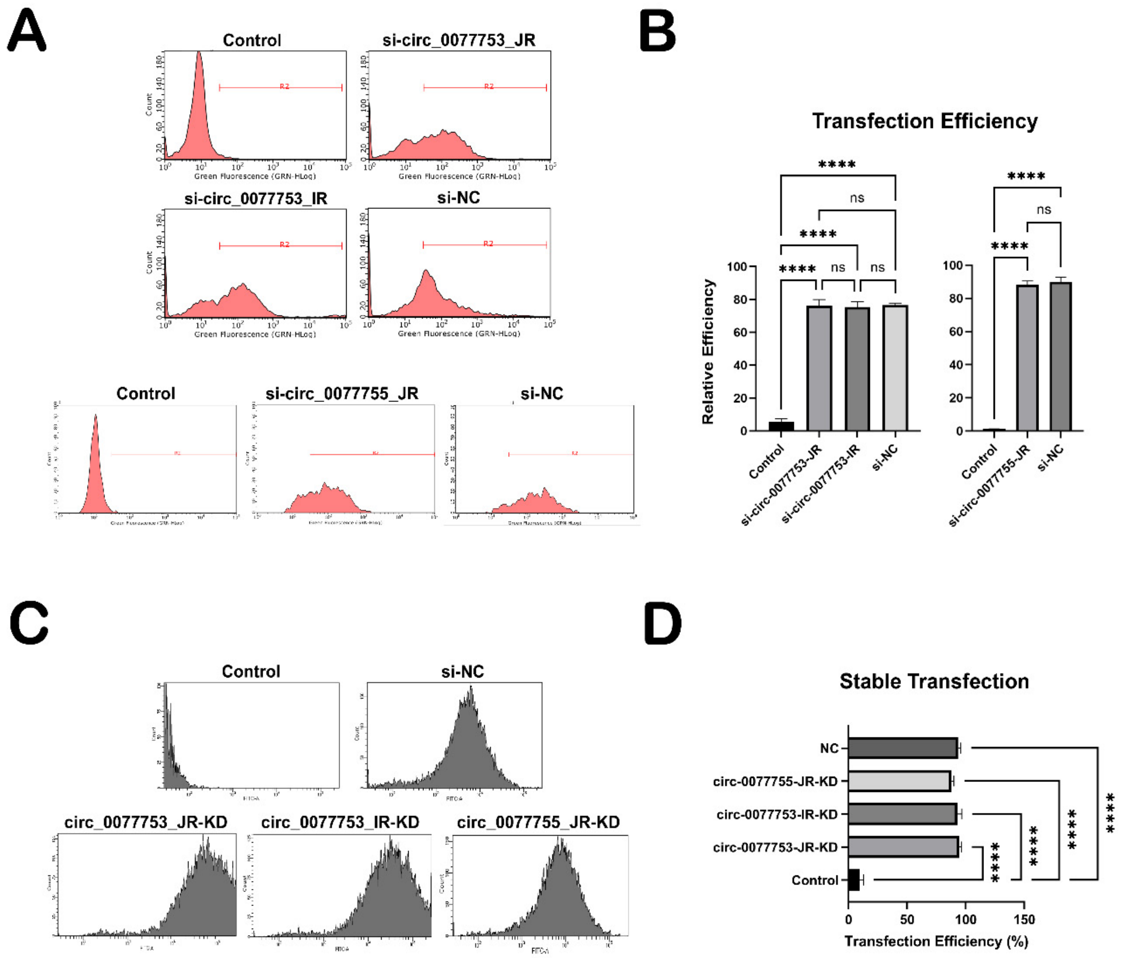

Figure S1. Transfection efficiency of transiently and stably transfected circRNA-knockdown S1 cells. (A) Representative flow cytometry profiles of transiently transfected S1 cells showing Control, si-circ_0077753-JR, si-circ_0077753-IR, si-circ_0077755-JR, and si-NC conditions. (B) Quantification of transient transfection efficiency for circ_753 and circ_755 knockdown experiments. (C) Representative flow cytometry profiles of stably transfected S1 cells, including Control, si-NC, circ_0077753-JR-KD, circ_0077753-IR-KD, and circ_0077755-JR-KD conditions. (D) Quantification of stable transfection efficiency for the corresponding knockdown conditions. Data are presented as mean ± SEM (n = 3 biologically independent experiments). **P < 0.0001; ns, not significant.

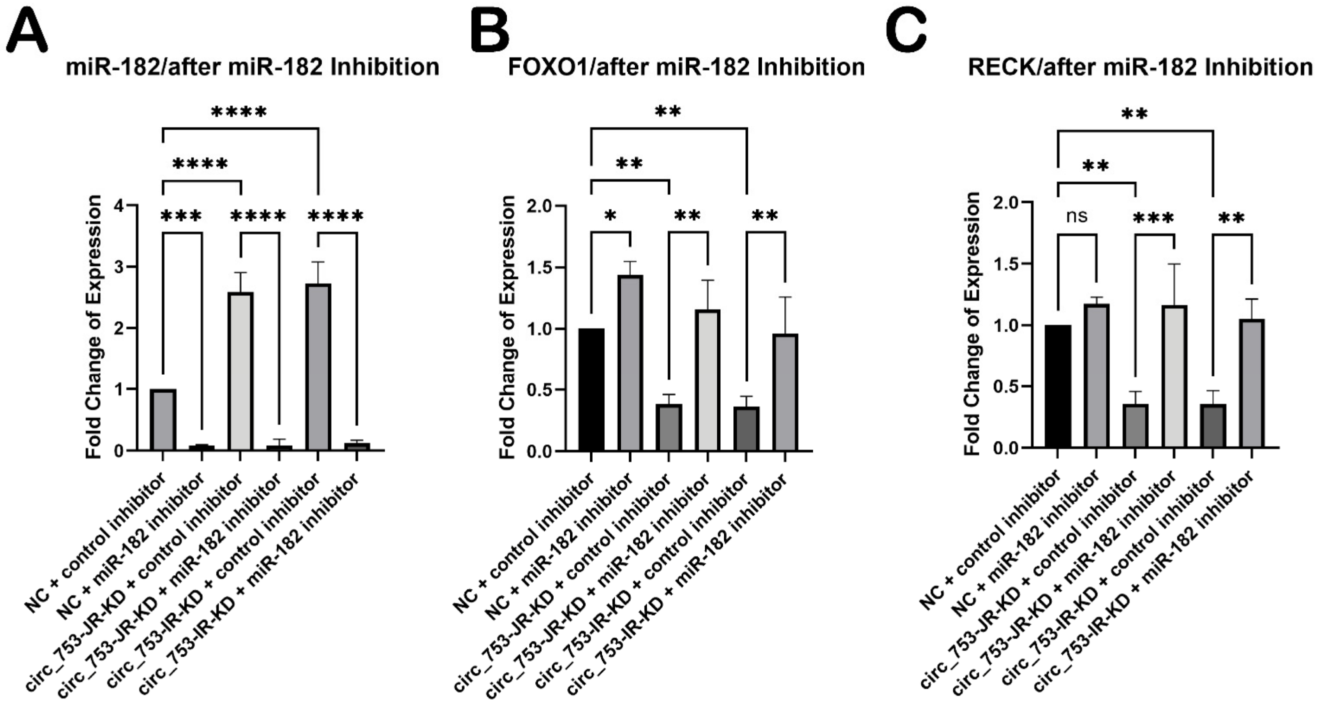

Figure S2. Effect of miR-182-5p inhibition on FOXO1 and RECK expression in circ_753-knockdown S1 cells. (A) RT-qPCR analysis of miR-182-5p expression in NC, circ_753-JR-KD, and circ_753-IR-KD cells treated with either a control inhibitor or a miR-182-5p inhibitor, confirming effective inhibition of miR-182-5p. (B, C) FOXO1 (B) and RECK (C) expression under the same conditions. miR-182-5p inhibition significantly increased FOXO1 and RECK expression in both circ_753-JR-KD and circ_753-IR-KD cells compared with their corresponding control-inhibitor conditions, supporting a contribution of miR-182-5p to the downstream effects associated with circ_753 depletion. Expression is presented as fold change relative to NC + control inhibitor, set to 1. Data are presented as mean ± SEM (n = 3 biologically independent experiments). **$P < 0.0001$; ns, not significant.
